# Photocatalytic and Electrocatalytic Generation of Hydrogen Peroxide: Principles, Catalyst Design and Performance

**DOI:** 10.1007/s40820-023-01052-2

**Published:** 2023-03-28

**Authors:** Yan Guo, Xili Tong, Nianjun Yang

**Affiliations:** 1 State Key Laboratory of Coal Conversion, Institute of Coal Chemistry, Chinese Academy of Sciences, Taiyuan, 030001 People’s Republic of China; 2 University of Chinese Academy of Sciences, Beijing, 100049 People’s Republic of China; 3 Institute of Materials Engineering, University of Siegen, 57076 Siegen, Germany; 4 Department of Chemistry, Hasselt University, 3590 Diepenbeek, Belgium; 5 IMO-IMOMEC, Hasselt University, 3590 Diepenbeek, Belgium

**Keywords:** H_2_O_2_ generation, Photocatalysts, Electrocatalysts, Reaction mechanisms

## Abstract

Hydrogen peroxide (H_2_O_2_) is a high-demand organic chemical reagent and has been widely used in various modern industrial applications. Currently, the prominent method for the preparation of H_2_O_2_ is the anthraquinone oxidation. Unfortunately, it is not conducive to economic and sustainable development since it is a complex process and involves unfriendly environment and potential hazards. In this context, numerous approaches have been developed to synthesize H_2_O_2_. Among them, photo/electro-catalytic ones are considered as two of the most promising manners for on-site synthesis of H_2_O_2_. These alternatives are sustainable in that only water or O_2_ is required. Namely, water oxidation (WOR) or oxygen reduction (ORR) reactions can be further coupled with clean and sustainable energy. For photo/electro-catalytic reactions for H_2_O_2_ generation, the design of the catalysts is extremely important and has been extensively conducted with an aim to obtain ultimate catalytic performance. This article overviews the basic principles of WOR and ORR, followed by the summary of recent progresses and achievements on the design and performance of various photo/electro-catalysts for H_2_O_2_ generation. The related mechanisms for these approaches are highlighted from theoretical and experimental aspects. Scientific challenges and opportunities of engineering photo/electro-catalysts for H_2_O_2_ generation are also outlined and discussed.

## Highlights

- Basic principles of photo- and electro-catalytic hydrogen peroxide generation.
- Recent progresses on the design, performance and mechanisms of photo- and electro-catalysts for hydrogen peroxide generation.
- Scientific challenges and prospects of engineering photo- and electro-catalysts for hydrogen peroxide production.

## Graphical abstract

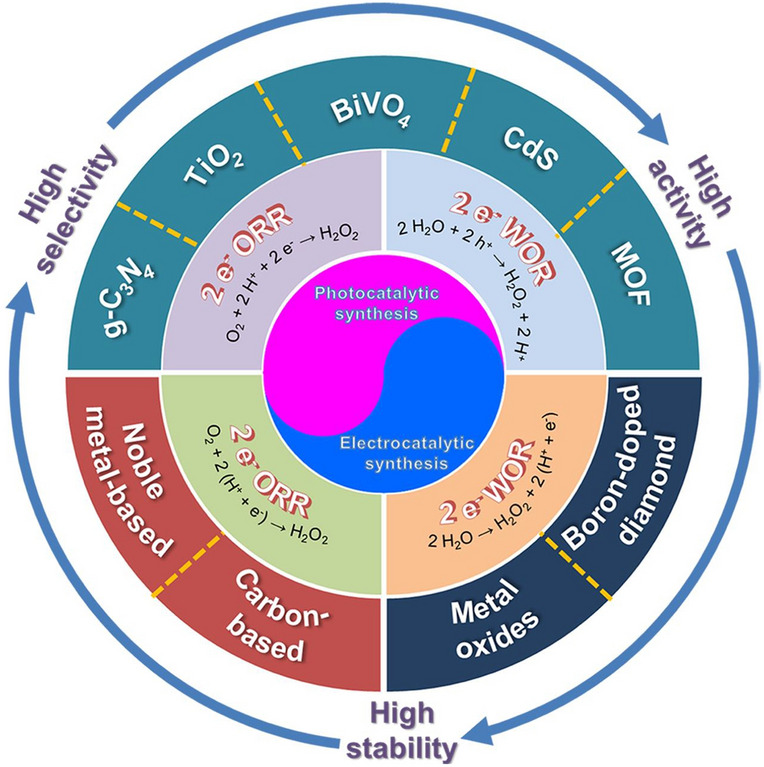

## Introduction

Hydrogen peroxide (H_2_O_2_) is a strong oxidant reagent compared with Cl-containing oxidants such as HOCl. This efficient and environmental-friendly chemical has been selected as one of the 100 most important chemical substances in the world [1, 2]. Owing to its strong oxidation capability, H_2_O_2_ has been widely used in a wide range of industrial and household applications, including wastewater treatment, chemical synthesis, industrial bleaching, energy storage, medical disinfection and a promising energy carrier in fuel cells [3–7]. It has been pointed out that H_2_O_2_ has advantages of the highest concentration of active oxygen (47.1 wt%) and the cleanest by-product (H_2_O) without carbon emission. These features make H_2_O_2_ a promising fuel alternative to H_2_ and O_2_ for one-chamber fuel cells without a membrane [8–10]. Although H_2_O_2_ fuel cells produce a bit lower theoretical output voltage (1.09 V) than H_2_/O_2_ fuel cells (1.23 V), the energy density of H_2_O_2_ (2.1 MJ kg^−1^ for 60% aqueous H_2_O_2_) is comparable to that of compressed hydrogen (3.5 MJ kg^−1^) [11–15]. In addition, H_2_O_2_ is a liquid fuel that can be fully soluble in water. It is thus more secure and facilitated to be transported and stored than the conventional H_2_. Accordingly, H_2_O_2_ has been predicted to be produced on a scale of approximately 5.7 million tons by 2027 in the global market, emphasizing its growing influence in the fields of sustainable energy [16].

**Fig. 1:**
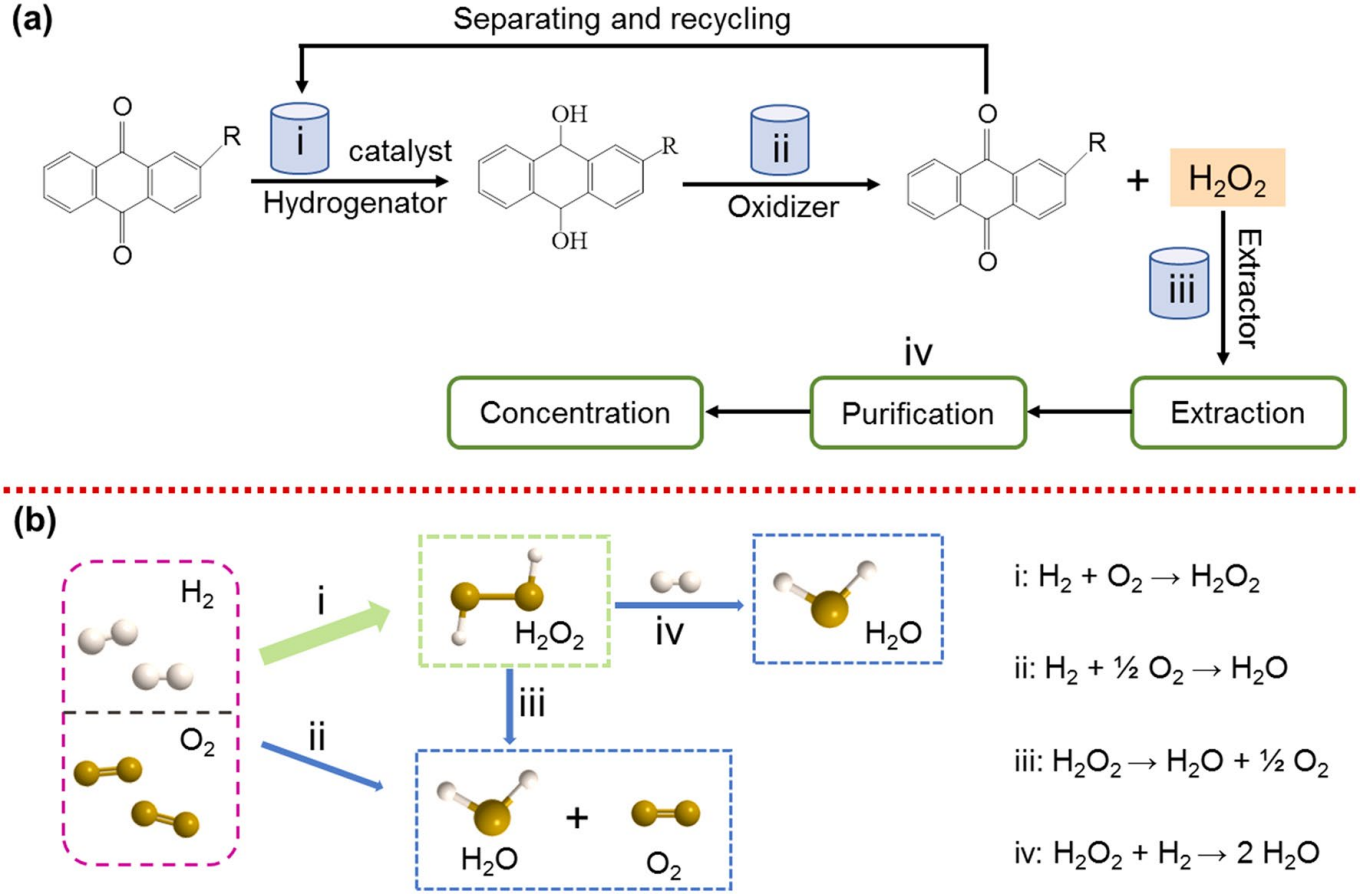
Schematic illustration of **a** the anthraquinone (AQ) oxidation process and **b** the direct synthesis from H_2_ and O_2_

The widely used method to synthesize H_2_O_2_ in the industry is anthraquinone (AQ) oxidation, which accounts for more than 95% of the total production of H_2_O_2_ [17]. Generally, the AQ oxidation process is a four-step cycle: (i) hydrogenation of AQ using a catalyst, (ii) oxidation of hydrogenated AQ to regenerate AQ and produce H_2_O_2_, (iii) extraction of H_2_O_2_ and (iv) purification concentration of H_2_O_2_. Therefore, this process requires a high energy demandingness and resource consumption and meanwhile produces many harmful by-products in series reactions (Fig. 1a) [18]. In this context, the pursuit of high concentration of H_2_O_2_ via AQ oxidation technology brings in more environmental pollutants and explosive substances, being threatened to the safety in fuel storage and transportation [19, 20]. To avoid these shortcomings of the AQ process, the direct preparation of H_2_O_2_ through the reaction from H_2_ and O_2_ appears more popular. It has been considered as a more energy-efficient and resource-efficient route alternative to the AQ oxidation (Fig. 1b) [21–24]. This partial hydrogenation reaction can be fully performed at low temperatures, if the highly efficient catalysts are designed and applied. It needs to be pointed out that this process usually selects expensive Pd-based materials as catalysts to limit competing side reactions [25, 26]. More seriously, it requires precise control of the ratio of H_2_ and O_2_ to leave for the explosive range, which unavoidably leads to low practice yields of the direct synthesis [27–29]. Another potential route to synthesize H_2_O_2_ is through a range of enzymatic catalytic processes in nature. It has been reported that some oxidase enzymes (e.g., glucose oxidase, D-amino acid oxidase and cholesterol oxidase) can selectively catalyze the corresponding native substrates to generate H_2_O_2_ [30–33]. Unfortunately, the inferior activity with a low value of turnover frequency occurs in these enzymatic catalytic reactions, which has impeded the rapid development of such biosynthesis of H_2_O_2_ [34, 35]. Consequently, environmental-friendly, efficient, safe and convenient alternative routes still need to be explored for H_2_O_2_ production.

In recent years, photo/electro-catalytic synthesis of H_2_O_2_ has attracted extensive attention. Comparison with the aforementioned three routes, these new approaches are more economical and environmental-friendly [36–39]. Belonging to direct synthesis methods, they can be realized using the abundant H_2_O and O_2_ on the earth as reactants, but are easier to be handled with lower operating risks [40, 41]. In addition, they can take full advantages of clean and renewable energy sources (e.g., solar energy, wind energy) in a sustainable fashion. To obtain efficient photo/electro-catalytic conversion efficiency, high-performance catalyst platforms need to be established. In other words, the design of advanced photo/electro-catalysts plays a vital role in such reaction conversion [42, 43]. From both fundamental and practical viewpoints, the rational design and controllable synthesis of various photo/electro-catalysts are thus of great importance for H_2_O_2_ synthesis.

In this regard, many efforts have been made in the design and synthesis of effective photo/electro-catalysts for active and selective generation of H_2_O_2_. It is therefore extremely significant to summarize the progress and state-of-art about design, characterization and application of developed photo/electro-catalysts for active and selective production of H_2_O_2_. This review article provides a comprehensive account of the development of photo/electro-catalysts toward H_2_O_2_ generation, covering related synthesis mechanisms, state-of-the-art catalysts/materials together with their performance evaluation criterion and typical engineering strategies for H_2_O_2_ formation. The future perspectives of photo/electro-catalyst design for H_2_O_2_ production are discussed and outlined.

## Mechanisms of Catalytic H_2_O_2_ Synthesis

### Photocatalytic Mechanisms of H_2_O_2_ Synthesis

The best advantage of photocatalytic synthesis is the direct utilization of renewable and sustainable solar energy to synthesize various products or degrade series of pollutes [44–46]. Most of recent photocatalytic synthesis studies focus on photocatalytic reactions on the semiconductors. During a typical photocatalytic process of H_2_O_2_ synthesis, three consecutive fundamental steps undergo on the photocatalysts [47]. In the first step, a semiconductor photocatalyst absorbs the excitation light, of which energy is greater than the band gap of the used photocatalyst, to create the negatively charged electron (e^−^) on its conduction band (CB), accompanying positively charged hole (h^+^) on its valence band (VB). In the second step, these photoinduced charge carriers (namely both e^−^ and h^+^) in the interior are separated and diffuse into the surface of the photocatalyst. Finally, they react with H_2_O and O_2_ to generate H_2_O_2_ via different redox pathways. Some recombine with each other and do not participate in any chemical reactions.

**Fig. 2:**
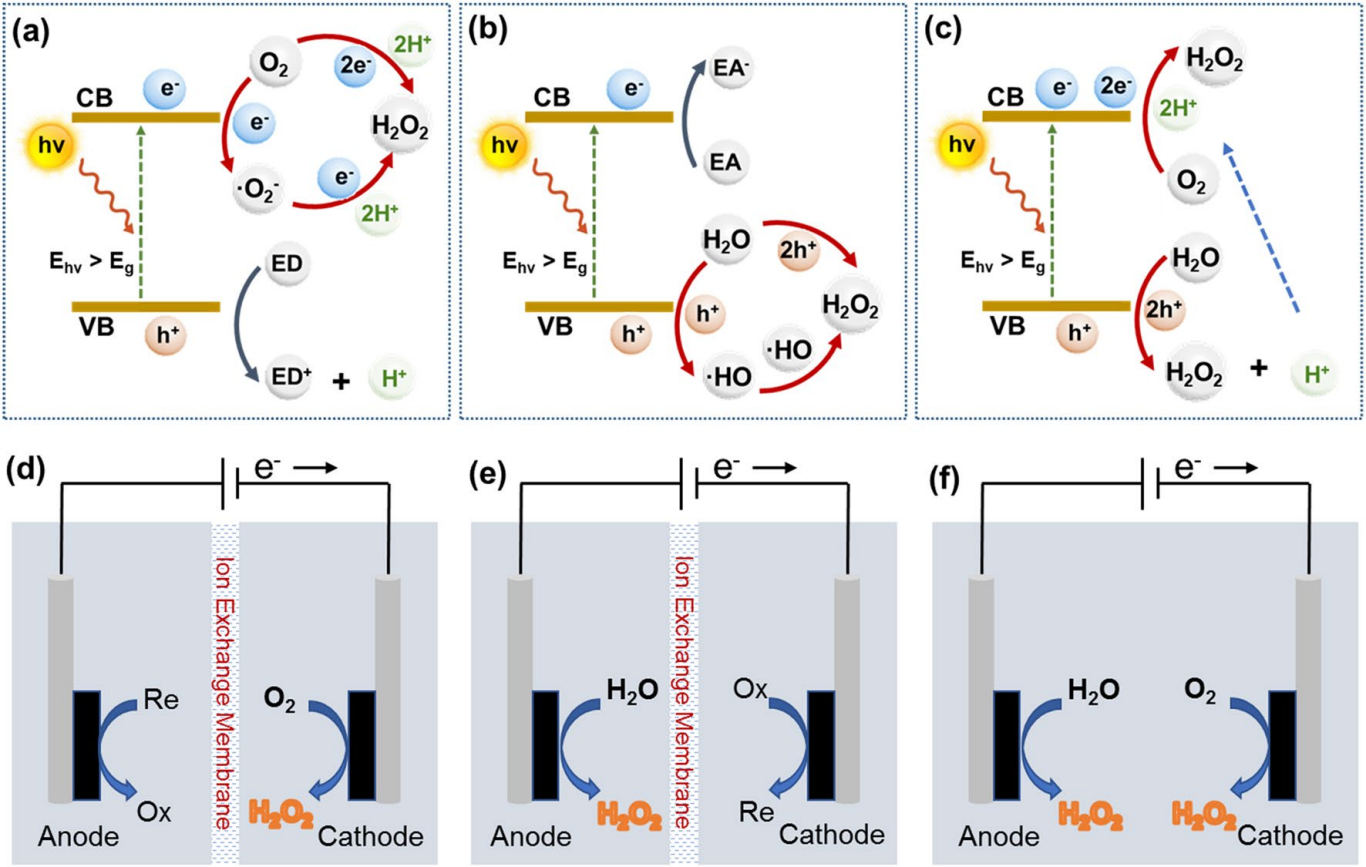
Simplified schematic of photocatalytic H_2_O_2_ generation from **a** ORR, **b** WOR and **c** dual-channel pathway. Simplified schematic of electrocatalytic H_2_O_2_ production from **d** ORR, **e** WOR and **f** dual-channel pathway

In the second step of a photocatalytic process of H_2_O_2_ synthesis, either oxygen reduction reaction (ORR) or water oxidation reaction (WOR) is involved [15]. As for the process of ORR (Fig. 2a), there are two potential mechanisms: indirect two-step single-electron (O_2_ → ·O_2_^−^ → H_2_O_2_) and direct one-step two-electron (O_2_ → H_2_O_2_) pathways. For example, the photons are absorbed by the semiconductor photocatalyst to excite the electrons in its VB to its CB, while holes are remained in its VB at the first step. Subsequently, the holes (h^+^) oxidize H_2_O to produce O_2_ (Eq. 1). In the indirect two-step single-electron pathway, one formed O_2_ molecule reacts with one electron (e^−^) to form O_2_^·−^ in the CB of the photocatalyst (Eq. 2a), which spontaneously combines H^+^ to generate a HO_2_^·−^ intermediate (Eq. 2b). This radical can also get one electron to produce HO_2_^−^ intermediate via another one-electron reduction reaction pathway (Eq. 2c). Finally, the synthesized HO_2_^−^ species reacts with H^+^, leading to H_2_O_2_ evolution in the form of Eq. 2d. In the direct one-step two-electron pathway, one formed O_2_ molecule directly reacts with two electrons (e^−^), resulting in the formation of H_2_O_2_, as demonstrated in Eq. 3.

1 $${\text{2 H}}_{{2}} {\text{O }} + {\text{ 4 h}}^{ + } \to {\text{O}}_{{2}} + {\text{ 4 H}}^{ + }$$

2a $${\text{O}}_{{2}} + {\text{ e}}^{ - } \to {\text{O}}_{{2}}^{\cdot- }$$

2b $${\text{O}}_{{2}}^{\cdot- } + {\text{ H}}^{ + } \to {\text{HO}}_{{2}}^{\cdot- }$$

2c $${\text{HO}}_{{2}}^{ \cdot- } + {\text{ e}}^{ - } \to {\text{HO}}_{{2}}^{ - }$$

2d $${\text{HO}}_{{2}}^{ - } + {\text{ H}}^{ + } \to {\text{H}}_{{2}} {\text{O}}_{{2}}$$

3 $${\text{O}}_{{2}} + {\text{ 2 H}}^{ + } + {\text{ 2 e}}^{ - } \to {\text{H}}_{{2}} {\text{O}}_{{2}}$$

The mechanism of H_2_O_2_ synthesis via the WOR (Fig. 2b) can be divided two pathways: direct one-step (H_2_O → H_2_O_2_) and indirect two-step (H_2_O → ^·^OH → H_2_O_2_) approaches. In the case of direct one-step process, one H_2_O molecule is directly oxidized by two holes to generate one H_2_O_2_ through the one-step reaction (Eq. 4). Regarding indirect two-step WOR, H_2_O is firstly be oxidized and the ^·^OH intermediate is formed (Eq. 5a). These intermediates react with each other to produce H_2_O_2_ (Eq. 5b).

4 $${\text{2 H}}_{{2}} {\text{O }} + {\text{ 2 h}}^{ + } \to {\text{H}}_{{2}} {\text{O}}_{{2}} + {\text{ 2 H}}^{ + }$$

5a $${\text{H}}_{{2}} {\text{O }} + {\text{ h}}^{ + } \to^{ \cdot } {\text{OH }} + {\text{ H}}^{ + }$$

5b $${2}^{ \cdot } {\text{OH}} \to {\text{H}}_{{2}} {\text{O}}_{{2}}$$

It is acknowledged that the process of photocatalytic H_2_O_2_ synthesis might simultaneously occur through both the ORR and WOR mechanisms (Fig. 2c). However, these strategies have the unavoidable drawbacks [48, 49]. Firstly, the solubility of O_2_ in liquid phase is low, limiting related reaction rates. Secondly, the recombination of electrons and holes is fast, reducing dramatically the efficiency of electron utilization. Thirdly, thermodynamic feasibility of the 4e^−^ pathway is greater than of the 2e^−^ pathway, leading to low selectivity of such processes. Therefore, it is necessary to design advanced photocatalysts to overcome these shortcomings.

The apparent quantum yield (AQY), defined as the ratio of the electron number in generating H_2_O_2_ molecule and the incident photons at a given wavelength, is an important indicator to evaluate the photocatalytic efficiency of various photocatalysts. It is frequently calculated using Eq. 6 [20].

6 $${\text{AQY}}\left( \% \right) \, = \frac{{2n}}{{N_{{aph}} }} \times { 1}00\%$$

where *n* is the electron amount of produced H_2_O_2_, *N*_aph_ is the number of incident photons shined on the photocatalysts.

In the section of discussion of photocatalysts (Chapter 3), we summarize recent progress and achievements on representative photocatalysts for H_2_O_2_ synthesis, in terms of their morphology and electronic band structures as well as their corresponding visible light-driven performance (e.g., activity, selectivity and stability) for H_2_O_2_ production.

### Electrocatalytic Mechanisms of H_2_O_2_ Synthesis

Electrocatalytic synthesis of H_2_O_2_ via the oxygen electrochemistry has been widely considered as an attractive route for on-site production of H_2_O_2_. Such an approach has effectively addressed the disadvantages of the AQ process and the possibility to be coupled with sustainable energy sources [50, 51]. Note that there are two direct pathways to produce H_2_O_2_, including two-electron oxygen reduction reaction (2e^−^ ORR, Fig. 2d) and two-electron water oxidation reaction (2e^−^ WOR, Fig. 2e) [52–54]. As for the 2e^−^ ORR process, the reduction of O_2_ to H_2_O_2_ proceeds by a two-step consecutive reaction road. This process can be described as an overall electrochemical reaction (Eq. 7), where $E^{\theta }$ represents the standard thermodynamic equilibrium potential, which is calculated to 0.70 V versus reversible hydrogen electrode (RHE). In more detail, one O_2_ molecule diffuses to the active surface of an electrocatalyst and gets adsorbed. If it gains one electron from the electrode and further reacts with one H^+^ ion, the OOH* intermediate is generated (Eq. 7a, noted that * represents an unoccupied active site). Since the formed OOH* possesses high chemical reaction activity, it can combine with another H^+^ ion and one electron to produce H_2_O_2,_ accompanying with regeneration of the active site (Eq. 7b). Unfortunately, the OOH* intermediate can undergo a four-electron pathway to decompose and form *O and *OH intermediates, which forms H_2_O instead of H_2_O_2_ [55]. The overall electrochemical reaction for the 4e^−^ ORR process is depicted as Eq. 8, where $E^{\theta }$ is 1.23 *V*_RHE_. In the reaction mechanism, the as-formed OOH* intermediate in the first step (Eq. 8a) reacts with one H^+^ ion and electron to form O^*^ (Eq. 8b), which is further combined with another H^+^ ion and electron. The formation of OH* intermediate (Eq. 8c) in this step leads to the undesirable 4e^−^ ORR, which forms H_2_O, an alternative to H_2_O_2_ (Eq. 8d). Therefore, the OOH* intermediate plays a vital role in determining the 2e^−^ or 4e^−^ ORR pathways. For example, retaining the O–O bond in OOH*, meaning weaker oxygen binding energy, is favorable to improve the selectivity of H_2_O_2_ production [54, 55]. On the contrary, the stronger oxygen binding energy facilitates the 4e^−^ ORR pathway, resulting in the electrocatalytic synthesis of H_2_O. The 2e^−^ ORR:

7 $$\text{O}_{2} + 2( {{\text{H}}^{ + } + {\text{ e}}^{ - } } ) \to \text{H}_{2} {\text{O }}_{2} \;\;\;E^{\theta } = 0.7{\text{ V}}_{\text{ RHE}}$$

7a $$^{*} {\mkern 1mu} + {\text{ O}}_{2} + {\mkern 1mu} \left( {{\text{H}}^{ + } + {\text{ e}}^{ - } } \right) \to {\text{OOH}}^{*}$$

7b $${\text{OOH}}^{*} \, + \, \left( {{\text{H}}^{ + } + {\text{ e}}^{ - } } \right) \to {\text{H}}_{{2}} {\text{O}}_{{2}} + \, ^{*}$$

The 4e^−^ ORR:

8 $$\text{O}_{2} \; + \;4(\text{H}^{ + } + e^{ - } ) \to 2\text{H}_{2} {\text{O }}\;\;\;E^{\theta } = 1.23\;{\text{ V}}_{\text{ RHE}}$$

8a $$^{*} \, + {\text{ O}}_{{2}} + \, \left( {{\text{H}}^{ + } + {\text{ e}}^{ - } } \right) \to {\text{OOH}}^{*}$$

8b $${\text{OOH}}^{*} \, + \, \left( {{\text{H}}^{ + } + {\text{ e}}^{ - } } \right) \to {\text{H}}_{{2}} {\text{O }} + {\text{ O}}^{*}$$

8c $${\text{O}}^{*} \, + \, \left( {{\text{H}}^{ + } + {\text{ e}}^{ - } } \right) \to {\text{OH}}^{*}$$

8d $${\text{OH}}^{*} \, + \, \left( {{\text{H}}^{ + } + {\text{ e}}^{ - } } \right) \to {\text{H}}_{{2}} {\text{O }} + \, ^{*}$$

Similar as photocatalytic process of H_2_O_2_ synthesis, both ORR and WOR might simultaneously occur in electrocatalytic process (Fig. 2f).

To evaluate the catalytic selectivity in the electrocatalytic H_2_O_2_ synthesis via the ORR approach, the number of electrons transferred (*n*) needs to be calculated by use of the rotating ring-disk electrode (RRDE) or rotating disk electrode (RDE) technique. For the RRDE measurements, linear sweep voltammetry (LSV) is generally performed on the disk electrode, while a constant potential (e.g., 1.2 *V*_RHE_) is applied on the ring electrode. When H_2_O_2_ is produced on the disk, it can diffuse to the ring then be detected. Equation 9 has been often employed to calculate the *n* value, where* I*_D_ is the current on the disk electrode, *I*_R_ is the current at the ring electrode, and *N* is the efficiency of H_2_O_2_ collection. The *N* value can be obtained using Eq. 10. For the RDE measurements, the Koutecky–Levich (K–L) equation is applied to calculate *n* (Eq. 11), where *I* is the measured steady-state current derived from the ORR (mA cm^−2^), *I*_K_ is the kinetic current of the reaction with active substances on the electrode surface, *F* is the Faraday constant (96,500 C mol^−1^), *A* is the geometric area of the work electrode, *D*_0_ is the diffusion coefficient of O_2_ in the electrolyte (cm^2^ s^−1^), *ω* is the angular rotation speed (*ω* = 2π*N*, *N* is the linear rotation speed), *ν* is the kinematic viscosity of the electrolyte (cm^2^ s^−1^), ${C}_{{O}_{2}}$ is the saturated concentration of O_2_ in the solution (mol cm^−3^).

9 $$n\; = \;4\;\frac{{I_{D} }}{{I_{D } + {\raise0.7ex\hbox{${I_{R} }$} \!\mathord{\left/ {\vphantom {{I_{R} } N}}\right.\kern-0pt} \!\lower0.7ex\hbox{$N$}} }}$$

10 $${\text{ N}}\;{ = }\;{ - }\frac{{I_{R} }}{{I_{D} }}$$

11 $$\frac{1}{I}\; = \;\frac{1}{{I_{K} }}\; + \;\frac{1}{{0.620nFAD_{0}^{2/3} {\upomega }^{1/2} {\upnu }^{ - 1/6} C_{{O_{2} }} }}$$

Using as-obtained *n* value, the selectivity of H_2_O_2_ production can be calculated according to Eq. 12.

12 $${\text{H}}_{2} {\text{O}}_{2}\, \left( {\text{\% }} \right){ } = { }\frac{4 - n}{2}$$

Another promising route for electrocatalytic synthesis of H_2_O_2_ is the 2e^−^ WOR pathway, which can be depicted in an overall electrochemical reaction (Eq. 13) with $E^{\theta }$ of 1.76 *V*_RHE_. In this process, one H_2_O molecule is firstly electrooxidized by active sites on the electrode to form OH^*^, providing one electron and H^+^ ion (Eq. 13a). Another H_2_O molecule goes through the same reaction (Eq. 13b). Two OH^*^ then combine with each other, leading to the production of H_2_O_2_ (Eq. 13c). Similar with the case in ORR, the 2e^−^ WOR pathway faces the influence from the 4e^−^ WOR pathway (Eq. 14), where the thermodynamically equilibrium potential (1.2 *V*_RHE_) is lower than that in the 2e^−^ WOR pathway [41, 56]. In more detail, the OH^*^ radicals generated in the first step (Eq. 14a) are further electrooxidized, resulting in the formation of O^*^ intermediate (Eq. 14b). These O^*^ intermediates then lose one electron to form OOH^*^ intermediate (Eq. 14c). Finally, the OOH^*^ intermediate converts into O_2_ molecular to complete this 4e^−^ WOR process (Eq. 14d).

The 2e^−^ WOR:

13 $$2{\text{H}}_{2} {\text{O}} \to {\text{H}}_{2} {\text{O}}_{2} + 2({\text{H}}^{ + } + {\text{e}}^{ - } )\,{\text{E}}^{\theta } = 1.76\,{\text{ V}}_{\text{ RHE}}$$

13a $$^{*} \, + {\text{ H}}_{{2}} {\text{O}} \to {\text{OH}}^{*} \, + \, \left( {{\text{H}}^{ + } + {\text{ e}}^{ - } } \right)$$

13b $$^{*} \, + {\text{ H}}_{{2}} {\text{O}} \to {\text{OH}}^{*} \, + \, \left( {{\text{H}}^{ + } + {\text{ e}}^{ - } } \right)$$

13c $${\text{OH}}^{*} \, + {\text{ OH}}^{*} \to {\text{H}}_{{2}} {\text{O}}_{{2}}$$

The 4e^−^ WOR:

14 $$2{\text{ H}}_{{2}} {\text{O}} \to {\text{O}}_{2} + 4({\text{H}}^{ + } + {\text{ e}}^{ - } )\,{\text{E}}^{\theta } = 1.23\,{\text{ V}}_{\text{ RHE}}$$

14a $$^{*} \, + {\text{ H}}_{{2}} {\text{O}} \to {\text{OH}}^{*} \, + \, \left( {{\text{H}}^{ + } + {\text{ e}}^{ - } } \right)$$

14b $${\text{OH}}^{*} \to {\text{O}}^{*} \, + \, \left( {{\text{H}}^{ + } + {\text{ e}}^{ - } } \right)$$

14c $${\text{O}}^{*} \to {\text{OOH}}^{*} \, + \, \left( {{\text{H}}^{ + } + {\text{ e}}^{ - } } \right)$$

14d $${\text{OOH}}^{*} \to ^{*} \, + {\text{ O}}_{{2}} + \, \left( {{\text{H}}^{ + } + {\text{ e}}^{ - } } \right)$$

The key factor to dominate the H_2_O_2_ synthesis via the 2e^−^ WOR pathway is thus the appropriate interaction between the catalytic surface and O intermediates (e.g., ^*^O, ^*^OH and ^*^OOH) [57]. Specifically, too strong OH binding promotively oxidizes ^*^OH to ^*^O, further to ^*^OOH, thereby prompting the undesirable 4e^−^ pathway to generate O_2_. On the other hand, too weak binding improves the selectivity of 2e^−^ WOR, but inevitably causes high kinetic barriers to hydrolysis and significantly slows down the reaction rate. It is disappointed that H_2_O_2_ produced in the 2e^−^ WOR process is not chemically stable. The undesirable disproportionation (2 H_2_O_2_ → 2 H_2_O + O_2_) or homolysis (H_2_O_2_ → 2 ·OH) spontaneously arises, decreasing the selectivity of H_2_O_2_ production. Therefore, the electrocatalysts with proper binding energies are highly pursued to boost 2e^−^ pathway toward H_2_O_2_ production.

In general, the selectivity of H_2_O_2_ generation via H_2_O electrooxidation is defined using the term of Faradaic Efficiency (%FE) (Eq. 15).

15 $$\% {\text{FE }} = \frac{{Q_{{H_{2} O_{2} }} }}{{Q_{total} }}$$

where ${Q}_{{H}_{2}{O}_{2}}$ is the charge that is used to produce H_2_O_2_, and ${Q}_{total}$ is the total charge passed during the whole process for water electrooxidation. In the section of the discussion of electrocatalysts (Chapter 4), we will summarize and evaluate research progress on representative electrocatalysts for synthesizing H_2_O_2_, in terms of the morphology, structure, composition as well as the corresponding activity, selectivity and stability.

It should be pointed out that the detection of the H_2_O_2_ concentration in the electrolyte is very important for the practical applications. Such a concentration is generally determined by the following methods, including [58]: (1) titration using potassium permanganate (KMnO_4_) or cerium sulfate [Ce(SO_4_)_2_], (2) colorimetric change from Fe^2+^ to Fe^3+^, (3) colorimetric determination using titanium (IV) sulfate and (4) high-performance liquid chromatography (HPLC). To separate the generated H_2_O_2_ from the electrolyte, the oil–water two-phase systems or distillation can be applied, although few reports have focused on such an issue.

## Photocatalytic H_2_O_2_ Synthesis

### Graphitic Carbon Nitride

Graphitic carbon nitride (g-C_3_N_4_) possesses a graphitic stacking of structure aromatic molecules with an alternating arrangement of earth-abundant carbon and nitrogen elements. Since the pioneering work of g-C_3_N_4_ for the promotion of photocatalytic hydrogen evolution under visible light irradiation, g-C_3_N_4_ has received strongly attention [59]. Such a situation is derived from this metal-free polymer photocatalyst features intrinsic advantages, such as its suitable band gap (~ 2.7 eV) for visible light response, facile preparation, relatively higher conductivity, excellent chemical stability in strong acidic and alkali media and special electronic structure [60–64]. In this context, it has been frequently employed for various photocatalytic applications, such as CO_2_ reduction, H_2_ production, and especially for H_2_O_2_ production [65–70]. Especially, since the CB potential (−1.3 V) of g-C_3_N_4_ is more negative than the reduction potential of O_2_/H_2_O_2_ (0.695 V), g-C_3_N_4_ is thermodynamically promising to reduce O_2_ for the H_2_O_2_ production using visible light [71]. In addition, the VB potential of g-C_3_N_4_ (1.4 V) is lower enough to effectively prevent the oxidative decomposition of H_2_O_2_ [64]. For example, Shiraishi’s group employed g-C_3_N_4_ for photocatalytic H_2_O_2_ production with the selectivity higher than 90%. The generated intermediate generated on the g-C_3_N_4_ surface 1,4-endoperoxide promotes the two-electron ORR pathway to produce H_2_O_2_ [68]. However, it must stress that the pristine g-C_3_N_4_ has many drawbacks that limit its photocatalytic performance toward the H_2_O_2_ production, covering its small specific surface area, weak ability to capture visible light and low chemical adsorption capacity of O_2_ on its surface [72, 73]. To overcome these shortcomings, ultra-thin g-C_3_N_4_ nanoplates and hexagonal rosettes of g-C_3_N_4_ have been prepared, which showed remarkable photocatalytic activity in the H_2_O_2_ production, stemming from their high surface area, rich active sites and strong light-harvesting capability [74, 75]. Unfortunately, the overall photo-conversion efficiencies of these g-C_3_N_4_ nano-photocatalysts are still unsatisfactory, due to rapid recombination of photogenerated carriers. Therefore, it is necessary to introduce organic scavengers into these nano-photocatalysts (e.g., ultra-thin g-C_3_N_4_ nanoplates and hexagonal rosettes of g-C_3_N_4_) to consume holes in the process of H_2_O_2_ production [70, 76]. In short, the efficiency for visible-light excitation, the separation of photogenerated carriers and photo-reaction kinetics of g-C_3_N_4_ photocatalysts must be further improved. In this regard, surface engineer of g-C_3_N_4_ photocatalysts has been proposed and developed to facilitate its performance with the enhanced the photocatalytic production efficiency of H_2_O_2_ on g-C_3_N_4_ photocatalysts. To optimize the photocatalytic H_2_O_2_ synthesis, the reported strategies cover manufacturing of surface defects, loading precious metal nanoparticles, constructing heterojunction composites, polyoxometalate hybridization and metal/non-metal element doping are performed (Table 1). In the following sessions, the details of these strategies are explained.

**Table 1:** Summary of the g-C_3_N_4_ photocatalysts for the H_2_O_2_ production

Catalyst	Organic sacrificial agent	Irradiation condition	H_2_O_2_ yield	Refs.
g-C_3_N_4_	Alcohol	*λ* > 420 nm	30 μmol (12 h)	[68]
reduced g-C_3_N_4_	–	*λ* > 420 nm	170 μmol L^−1^ h^−1^	[70]
AQ/U-POCN	Isopropanol	*λ* = 400–780 nm	75 μM h^−1^	[63]
m-CNNP	Isopropanol	*λ* = 400–700 nm	43.07 μmol g^−1^ h^−1^	[74]
CM-CN3	Ethanol	*λ* = 400–800 nm	150 μmol g^−1^ h^−1^	[75]
ACNT-5	–	simulated sunlight (AM 1.5 filter)	240.36 μmol h^−1^ g^−1^	[116]
PH-CN	Ethanol	*λ* = 400–800 nm	5.2 mmol L^−1^ (12 h)	[85]
DCN-15A	IPA	*λ* > 420 nm	12.1 μmol (2.5 h)	[79]
CN-ND	Ethanol	300 W xenon lamp	200 μM (1 h)	[86]
IO CN-Cv	EtOH	300W xenon lamp	325.74 μM (2 h)	[83]
NDCN	Isopropanol	*λ* ≥ 420 nm	476 μM g^−1^ h^−1^	[87]
NVCNS	IPA	100 mW cm^−2^Xe lamp	4413.1 μmol g^−1^ h^−1^	[117]
0.01% Au/CN	C_2_H_5_OH	*λ* > 420 nm	2027 μM (30 h)	[90]
Ag@U-g-C_3_N_4_-NS	–	100 mW cm^−2^	0.75 × 10^–6^ M min^−1^	[91]
Cu_2_(OH)_2_CO_3_/g-C_3_N_4_	–	simulated solar light source	8.9 mmol L^−1^ (6 h)	[98]
PI_5.0_-NCN	–	300W Xe lamp	120 μmol (120 min)	[97]
Bi_4_O_5_Br_2_/g-C_3_N_4_	–	*λ* > 420 nm	124 μM (60 min)	[99]
1.0 ZIS/CN	IPA	simulated solar (100 mW cm^−2^)	798 μmol h^−1^ g ^−1^	[118]
CN/rGO@BPQDs-0.04	–	*λ* = 420–780 nm	181.69 μmol L^−1^ (180 min)	[119]
3DOM g-C_3_N_4_-PW_11_	–	*λ* > 320 nm	14.4 μmol (360 min)	[105]
g-C_3_N_4_-SiW_11_	Methanol	sunlight irradiation (AM 1.5 filter)	91.4 μmol (360 min)	[106]
g-C_3_N_4_-PWO	–	*λ* ≥ 420 nm	11.8 μmol (240 min)	[107]
g-C_3_N_4_-CoWO	–	*λ* ≥ 420 nm	9.7 μmol h^−1^	[108]
Br doped g-C_3_N_4_	EDTA	*λ* = 400–800 nm	1.99 mmol L^−1^ (5 h)	[114]
Cu(2)-SCN	EDTA-2Na	*λ* = 400–800 nm	4.8 mmol L^−1^ (18 h)	[109]
K^+^/Na^+^-doped g-C_3_N_4_	–	*λ* = 400–800 nm	4.6 mmol L^−1^ (18 h)	[110]
KNiCN	–	*λ* ≥ 420 nm	398 μmol g^−1^ h^−1^	[111]
g-C_3_N_4_/BDI	2-PrOH	*λ* > 420 nm	41 μmol (48 h)	[120]
OCN	Isopropanol	100 mW cm^−2^	300 μmol (5 h)	[121]
AQ-augmented C_3_N_4_	–	simulated solar light (AM 1.5)	361 μmol g^−1^ h^−1^	[122]
g-C_3_N_4_-CNTs	Formic acid	*λ* ≥ 400 nm	130.2 μmol (240 min)	[123]
Au/C_3_N_4_	–	100 mW cm^−2^	1320 μmol L^−1^ (4 h)	[89]
SS-CN	–	300 W Xe lamp	566.69 μmol g^−1^ h^−1^	[124]
PT-g-C_3_N_4_	Ethanol	*λ* > 400 nm	27.07 μmol L^−1^ (60 min)	[125]
C_v_-gCN	–	*λ* > 420 nm	–	[126]
Ni-CAT-CN	–	*λ* ≥ 420 nm	1801 μmol h^−1^ g^−1^	[127]

#### Manufacturing Surface Defects

Vacancy defects are known to have the ability to capture photogenerated electrons/holes, thereby effective inhibiting the recombination of photogenerated electrons and holes [77–80]. Meanwhile, they can enhance the adsorption and activation of gas molecules (e.g., O_2_), due to their abundant local electrons for the adjustment of the electronic structure, thus facilitating a photocatalytic process [81, 82]. In general, two main types of defects appear in (or on) the g-C_3_N_4_ photocatalysts: carbon and nitrogen vacancies.

**Fig. 3:**
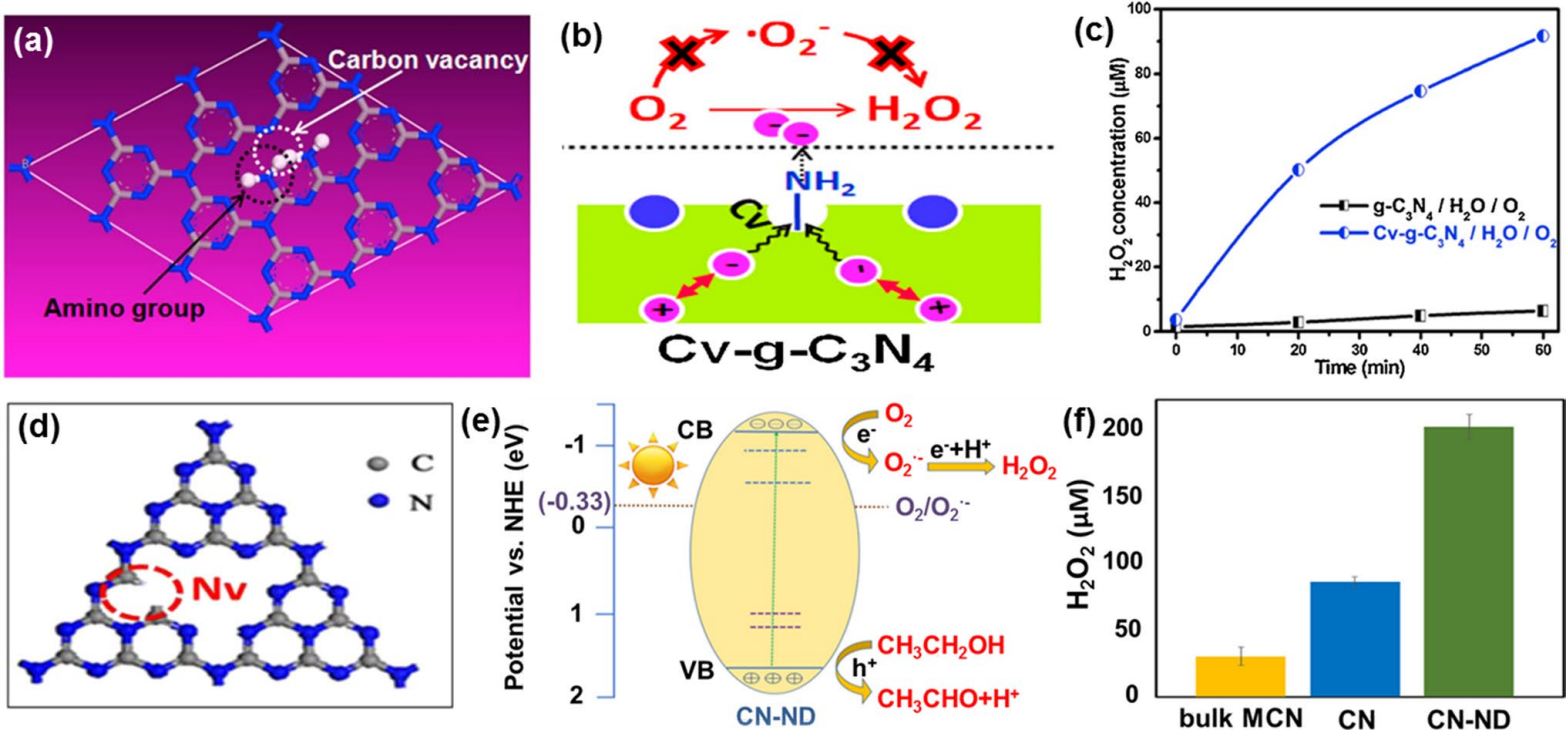
**a** Schematic illustration of g-C_3_N_4_ with carbon vacancy. **b** Mechanism diagram of H_2_O_2_ production in Cv-g-C_3_N_4_. **c** Concentration of generated H_2_O_2_ in g-C_3_N_4_ and Cv-g-C_3_N_4_ systems. Reproduced with permission from Ref. [71]. Copyright 2016 Elsevier. **d** Schematic illustration of g-C_3_N_4_ with nitrogen vacancies (CN-ND). **e** Proposed mechanism for H_2_O_2_ generation of CN-ND. **f** H_2_O_2_ production of different catalysts. Reproduced with permission from Ref. [86]. Copyright 2021 American Chemical Society

*Carbon vacancies* The defects derived from carbon element in/on g-C_3_N_4_ photocatalysts bring in carbon vacancies. They stand at mid-gap states between VB and CB and thus can capture more visible light [46]. Namely, the optical and electronic structures of g-C_3_N_4_ photocatalysts can be optimized. For instance, the introduction of carbon vacancies to the surface of g-C_3_N_4_ accompanied the appearance of amino group by simple calcination under argon atmosphere (Fig. 3a). In this case, the generated carbon vacancies (Cv-g-C_3_N_4_) reduce the band gap of g-C_3_N_4_, thereby expanding the absorption of visible light range and increasing excitable electron longevity [71]. Moreover, carbon vacancies offer more sites to adsorb molecular oxygen, beneficial for the transfer of electrons from g-C_3_N_4_ to the surface adsorbed O_2_. Furthermore, this Cv-g-C_3_N_4_ photocatalyst alters the H_2_O_2_ generation pathway: from a two-step single-electron indirect reduction pathway to a one-step two-electron direct one. This is attributed to generated amino groups around carbon vacancies (Fig. 3b). The activity of Cv-g-C_3_N_4_ for the reduction of O_2_ to H_2_O_2_ is 14-fold higher than that of a pure g-C_3_N_4_ photocatalyst under irradiation with visible light (Fig. 3c). Carbon vacancies (C_vs_) are then believed to produce more trapping sites, which retards the recombination of photogenerated electrons and holes, eventually resulting in an increased efficiency of the H_2_O_2_ generation [83]. Another representative case to strengthen the photocatalytic H_2_O_2_ synthesis was achieved via the creation of more vacancies, where an inverse opal (IO) structure was constructed on g-C_3_N_4_ accompanying with increased carbon vacancies to improve the separation ability of carriers. This IO g-C_3_N_4_ photocatalyst showed an outstanding H_2_O_2_ generation yield. For example, 325.74 μM was realized only after 2 h of visible light irradiation. The pathway of the H_2_O_2_ production was proposed as following: the electrons on the CB of this photocatalyst are feasible to reduce O_2_ to form H_2_O_2_ because its CB is more negative than that of O_2_/·O_2_^−^.

*Nitrogen vacancies* The defects from nitrogen element in/on g-C_3_N_4_ photocatalysts are called nitrogen vacancies. Similar to carbon vacancies, they can also facilitate the photocatalytic H_2_O_2_ synthesis. For example, nitrogen vacancies in g-C_3_N_4_ have been created via the formation of C≡N functional groups by means of thermal reduction treatment [70]. The as-formed nitrogen vacancies narrow down the band gap and make the band edge positively shift, thus enhancing the visible-light absorption and facilitating photocatalytic H_2_O_2_ synthesis [84]. Nitrogen vacancies in carbon nitride also effectively trap photoelectrons, boosting the reduction of N_2_ to ammonium ions [82]. For the photocatalytic H_2_O_2_ synthesis, nitrogen vacancies have been designed and embedded in the g-C_3_N_4_ catalyst (PH-CN) using a dielectric barrier discharge (DBD) plasma in H_2_ atmosphere. Since this photocatalyst improves the separation efficiency of the photogenerated carriers, it displays a H_2_O_2_ concentration of 5.2 mmol L^−1^, 13 times higher than that obtained on a pristine g-C_3_N_4_ photocatalyst under the identified conditions [85]. In this case, nitrogen vacancies are assumed not only to serve as active centers for the adsorption of reactive oxygen molecules, but also to alter the electronic band structures of g-C_3_N_4_ to harness more visible light in the photocatalytic reaction process. One recently reported the nanostructured g-C_3_N_4_ with nitrogen defects was adjusted by cyanuric acid-melamine supramolecular adducts (CN-ND) in the optimum range (Fig. 3d) [86]. Due to the narrowed band gap and the newly formed midgap states, the photocatalytic H_2_O_2_ production on CN-ND reached 200 μM under 1 h visible light irradiation, much higher than that obtained bulk g-C_3_N_4_ photocatalyst (35 μM) and the pristine nanostructured g-C_3_N_4_ photocatalyst (85 μM) (Fig. 3e, f). Again, such improved performance of photocatalytic H_2_O_2_ generation was attributed to these nitrogen vacancies since they promote the visible-light-harvesting capability and retard the recombination rate of photogenerated carriers. Meanwhile, the reaction mechanism of photocatalytic H_2_O_2_ production on nitrogen deficiency in carbon nitride has been investigated through coupling experiments with the aid of time-dependent density functional theory (TDDFT) and density functional theory (DFT) calculations [87]. It is disclosed that the introduced bicoordinated nitrogen vacancies play an important role in oxidation, reduction and charge recombination, which are beneficial to the generation of h^+^, ·O_2_^−^ and ^1^O_2_, respectively. Therefore, nitrogen vacancies in carbon nitride can modulate photocatalytic H_2_O_2_ generation.

#### Loading Precious Metal Nanoparticles

**Fig. 4:**
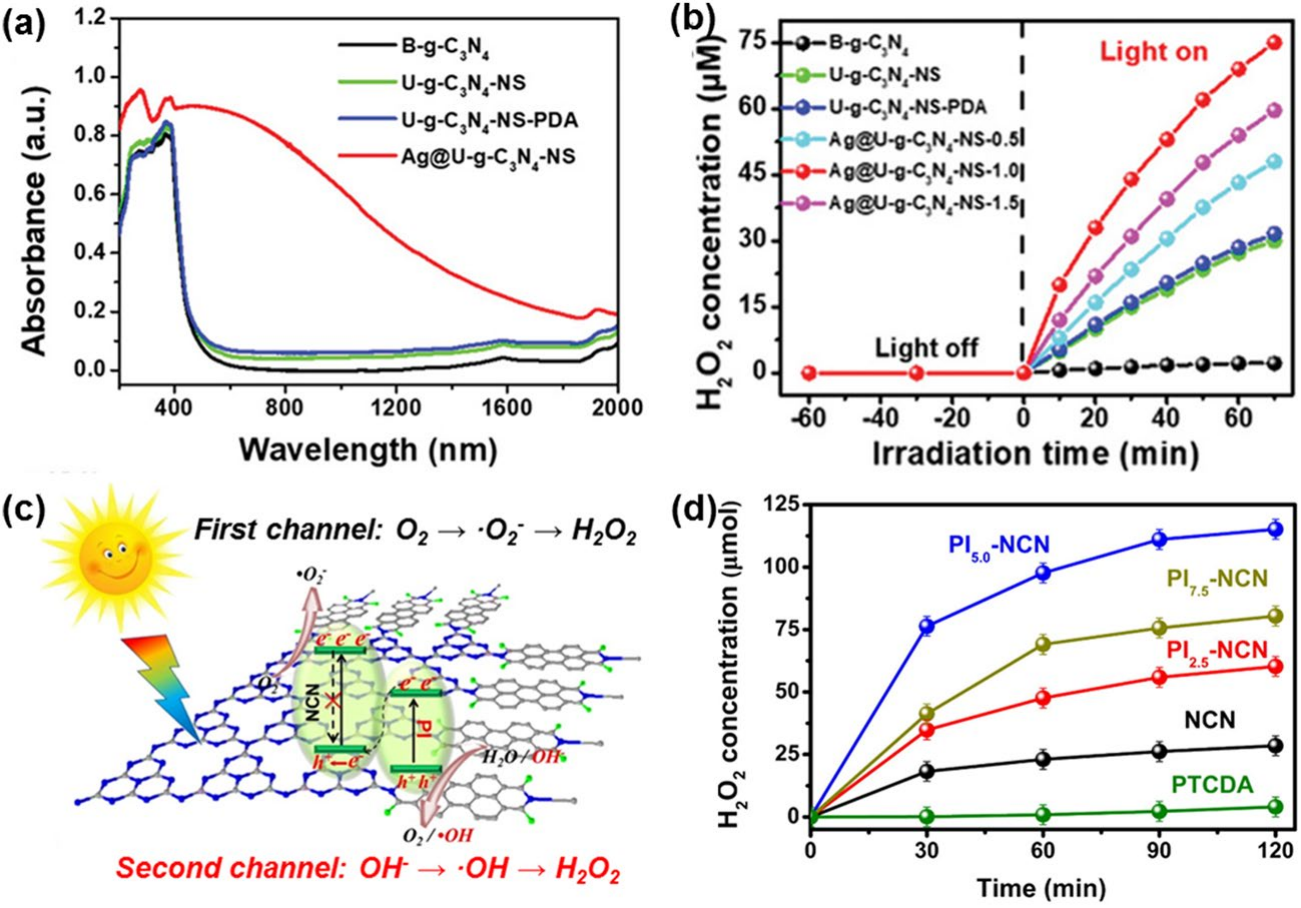
**a** UV-DRS absorption spectra of different samples. **b** H_2_O_2_ concentration of different catalysts. Reproduced with permission from Ref. [91]. Copyright 2019 Wiley–VCH Verlag GmbH & Co. KGaA, Weinheim. **c** Proposed mechanism of different sites on all-solid-state Z-scheme heterojunctions for photocatalytic production of H_2_O_2_. **d** Concentration of generated H_2_O_2_ in NCN and PIx-NCN systems. Reproduced with permission from Ref. [97]. Copyright 2017 Elsevier

The loading of precious metal nanoparticles (NPs) on g-C_3_N_4_ forms strong interaction between metallic NPs and g-C_3_N_4_, as derived from the delocalization of long electrons in the g-C_3_N_4_ matrix. This is one of the most effective approaches to strengthen the photocatalytic activity and selectivity of g-C_3_N_4_ since the photogenerated charge separation in such photocatalysts are accelerated, leading to boost photocatalytic reaction in the special pathways [88]. In addition, these photocatalysts suppress the H_2_O_2_ decomposition to obtain high yields during photocatalytic H_2_O_2_ synthesis. For example, Au NPs deposited on the surface of g-C_3_N_4_ by a carbon-layer-stabilized method exhibited a higher photocatalytic yield of H_2_O_2_ than the pristine g-C_3_N_4_ due to the increased removal of the generated H_2_O_2_ from the active reactive sites [89]. In another case, Au NPs were uniformly dispersed on g-C_3_N_4_ support (Au/CN) and further utilized to produce H_2_O_2_ under visible light irradiation [90]. Its outstanding photocatalytic yield of H_2_O_2_ was believed to result from its inertness to catalyze the H_2_O_2_ decomposition reaction. The use of 0.01% Au led to the maximum H_2_O_2_ production activity (2027 μM) with light irradiation for 30 h, superior to that using other NPs (e.g., Au, Ag, Pd and Pt) on g-C_3_N_4_. This high activity of 0.01% Au/CN was attributed to the rapidly reduced recombination of charge carriers. It was derived from the strong interaction between Au and g-C_3_N_4_, as testified by obviously quenched PL signal. Photocatalytic H_2_O_2_ synthesis for this Au/CN photocatalyst has been further revealed using *in-situ* electron spin resonance (ESR) technique, indicating so-called direct 2e^−^ reduction mechanism. With similar aim to extend light absorption region and effectively suppress the recombination of electron–hole pairs for photocatalytic H_2_O_2_ synthesis, Ag NPs have also been combined with g-C_3_N_4_ [91]. For example, Ag NPs have been loaded on ultrathin g-C_3_N_4_ nanosheets (U-g-C_3_N_4_-NS) by use of a post gas etching (PGE) technology. These Ag NPs that are uniformly deposited on the surface of single U-g-C_3_N_4_-NS layers (Ag@U-g-C_3_N_4_-NS) possess significantly stronger adsorption intensity in the 200–2000 nm range than the counterparts in their UV–vis diffuse reflectance spectrum (UV–DRS, Fig. 4a). It was suggested that such enhanced adsorption of Ag@U-g-C_3_N_4_-NS was attributed to the special structure of the U-g-C_3_N_4_-NS and the localized surface plasmon resonance (LSPR) effect from the Ag NPs. Effective separation of photogenerated carriers helped to adsorb light more efficiently, leading to their enhanced photocatalytic H_2_O_2_ activity. An optimal structure of the Ag@U-g-C_3_N_4_-NS-1.0 nanocomposite showed excellent photocatalytic activity of H_2_O_2_ production with a yield of 1.975 × 10^–6^ M min^−1^ under visible-light irradiation, even without any sacrificial carbon-containing organic electron donor (Fig. 4b).

#### Constructing Heterojunction Composites

Using g-C_3_N_4_ photocatalysts that contain N and/or C vacancies to prepare H_2_O_2_, the organic reagents (such as methanol and ethanol) are frequently added. They usually act as hole scavengers to improve the yield, but contaminate the product of H_2_O_2_, and further increase the cost. Note that the potential of VB of g-C_3_N_4_ (+1.57 V) is negative than that of ·OH/OH^−^ (+1.99 V) [92–94], the H_2_O_2_ synthesis is thus hard to be realized through photocatalytic H_2_O oxidation mechanism. Instead, a two-channel H_2_O_2_ generation approach (namely O_2_ reduction and hole oxidation OH^−^) on g-C_3_N_4_ is highly desirable. To overcome these issues, heterogeneous composites of g-C_3_N_4_ with other components were constructed. The separation of photogenerated carriers of these composites were enhanced by introduced electron traps, increased potential of VB and created additional active sites. On these composites, the H_2_O_2_ generation was achieved via the H_2_O oxidation pathway. More importantly, this strategy spatially isolates the oxidation and reduction reaction sites, bringing in minimized deactivation of catalytic ability of these photocatalysts. It has been reported that *Z*-scheme heterostructures with a wide range of light-trapping properties promoted the separation of photogenerated carriers but without the introduction of hole scavengers and thus rapidly improved the catalytic H_2_O_2_ production [95, 96]. For example, the combination g-C_3_N_4_ nanosheets (NCN) with perylene imides (PI) has been reported to form an all-solid-state *Z*-scheme heterojunction: a g-C_3_N_4_-based photocatalyst (PI_x_-NCN), where the excited electrons on the CB of PI are transferred to the VB of NCN, leading to retard recombination of photogenerated carriers (Fig. 4c) [97]. In this case, this PI_x_-NCN photocatalyst obviously enhanced the charge separation, resulting in an enhanced H_2_O_2_ yield where more electrons were found to take participate in producing H_2_O_2_ from O_2_ reduction. In addition, since the holes in the VB of PI moiety exhibited more positive potential (2.08 V) than that of NCN (1.63 V), the H_2_O_2_ generation was assumed to be realized through the direct 2e^−^ WOR approach. In other words, the application of scavengers was avoided here. The optimal PI_5.0_-NCN catalyst showed high activity for the H_2_O_2_ production: the generation of about 120 μmol H_2_O_2_ in 120 min under visible-light irradiation (Fig. 4d). Meanwhile, other *Z*-scheme heterojunction catalysts (e.g., Cu_2_(OH)_2_CO_3_/g-C_3_N_4_ [98] and Bi_4_O_5_Br_2_/g-C_3_N_4_ [99]) have been employed for the two-channel H_2_O_2_ production under visible-light conditions, thanks to their suitable CB and VB band structures for realizing completed ORR and WOR pathways.

#### Polyoxometalate Hybridization

**Fig. 5:**
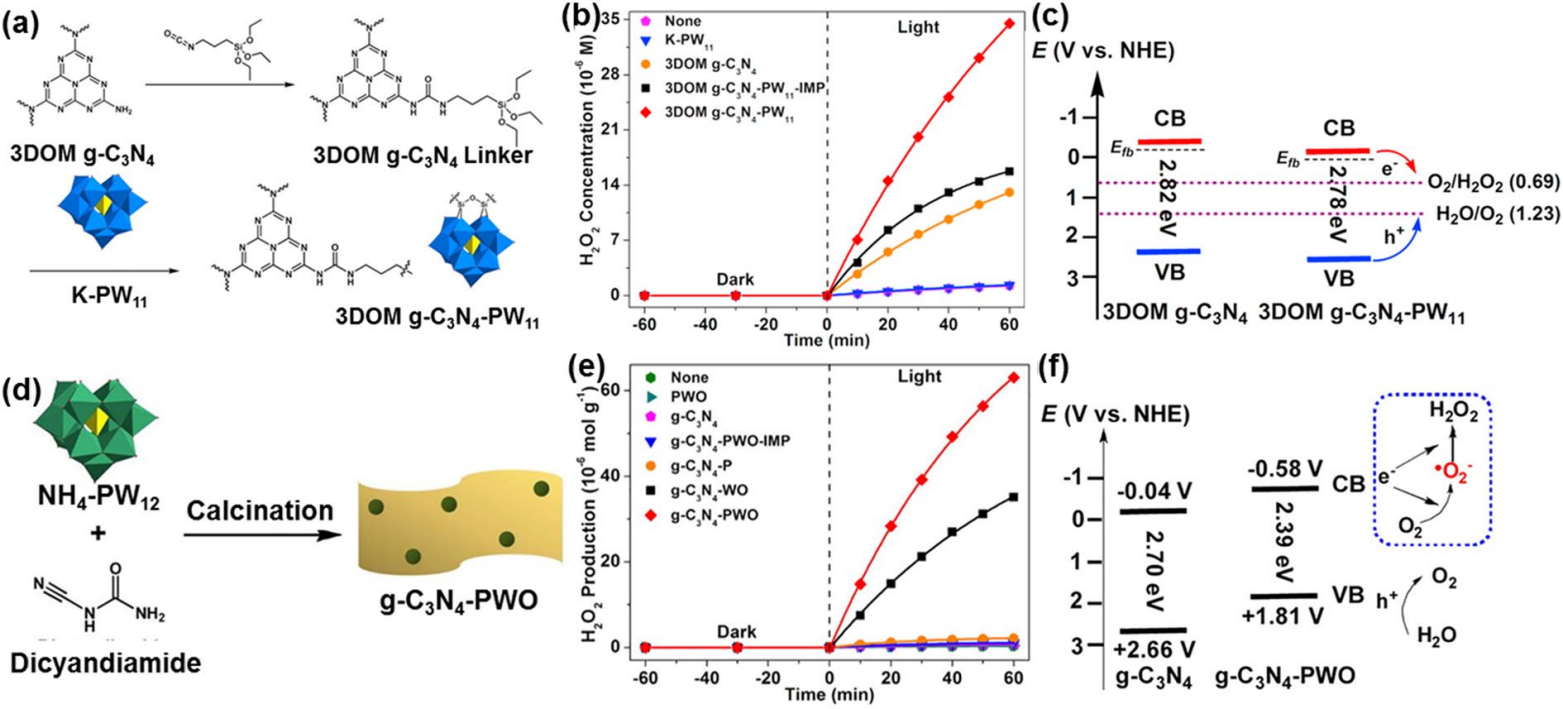
**a, d** Schematic illustration of the synthetic route to 3DOM g-C_3_N_4_-PW_11_ and g-C_3_N_4_-PWO. **b, e** H_2_O_2_ production over different catalysts in 60 min. **c, f** Scheme of energy levels and charge transfer pathways of 3DOM g-C_3_N_4_ and 3DOM g-C_3_N_4_-PW_11_, g-C_3_N_4_ and g-C_3_N_4_-PWO. Reproduced with permission from Refs. [105, 107]. Copyright 2017 Elsevier and 2018 Elsevier

Polyoxometalates (POMs) are commonly constructed using a basic unit of oxo-metal polyhedra of MO_x_, in which a hole center (O^−^) and a trapped electron center (M^n+^) (n = 5, 6) act as the electron acceptor and donor, respectively [100]. Once POMs are excited by visible light irradiation, they exhibit promising photocatalytic activities and stability. The reason behind is that the reduction of photogenerated-charge recombination is derived from the well-defined HOMO–LUMO band gaps. In this context, POMs have been widely applied in the field of photocatalysis, such as for water oxidation, alcohols oxidation, CO_2_ reduction and hydrogen evolution [101–104]. More interestingly, polyoxometalate hybridization strategy has been recently proposed, namely the formation of POM chemical bonds with g-C_3_N_4_, which was further utilized to improve the photocatalytic H_2_O_2_ synthesis. For instance, the POM cluster of [PW_11_O_39_]^7−^ (PW_11_) has been covalently combined with three dimensionally ordered macroporous graphitic carbon nitride (3DOM g-C_3_N_4_), applying for the photocatalytic H_2_O_2_ synthesis (Fig. 5a) [105]. The hybrid catalyst of 3DOM g-C_3_N_4_-PW_11_ produced 3.5 μmol H_2_O_2_ in 60 min irradiated by λ ≥ 320 nm light, where no organic electron donor was needed (Fig. 5b). Such a H_2_O_2_ yield was superior to that of the pure 3DOM g-C_3_N_4_ catalyst (1.3 μmol), the K-PW_11_ catalyst (< 0.1 μmol) and the 3DOM g-C_3_N_4_-PW_11_-IMP catalyst (1.5 μmol). It was explained that the covalent bonding of PW_11_ clusters with 3DOM g-C_3_N_4_ optimizes the VB and the CB of 3DOM g-C_3_N_4_-PW_11_ when compared with 3DOM g-C_3_N_4_. Such optimal band structures facilitate both the 2e^−^ WOR pathway and the 2e^−^ ORR pathway for the H_2_O_2_ production, accompanying with the decreased separation of photogenerated holes and electrons.

On the other hand, 3DOM g-C_3_N_4_-PW_11_ hybrid catalyst still has some defects. For instance, the number and reactivity of -NH_2_ groups of g-C_3_N_4_ need to be enhanced to further increase the covalent interaction between g-C_3_N_4_ and POMs. Moreover, the photoreduction ability of POMs needs to be improved to further enhance the selectivity of the 2e^−^ ORR pathway. In this context, the surface of g-C_3_N_4_ has been modified by its covalent binding with another POM cluster, [SiW_11_O_39_]^8−^ (SiW_11_) via thermal treatment of g-C_3_N_4_ mixed with urea [106]. The as-prepared g-C_3_N_4_ catalyst has a larger amount of more highly active-NH_2_ groups than 3DOM g-C_3_N_4_. Via an organic linker bride, the POM SiW_11_ cluster with more negative CB potential than PW_11_ was effectively covalently combined with g-C_3_N_4_. This g-C_3_N_4_-SiW_11_ greatly enhanced the adsorption and activation of O_2_, thereby promoting the two-electron reduction of O_2_ for the H_2_O_2_ production. It produced 17.8 μmol H_2_O_2_ in 60 min under simulated sunlight (AM 1.5) irradiation together with the selectivity as high as 80.1% in the presence of methanol. The high selectivity of O_2_ reduction to H_2_O_2_ was attributed to a positive shift of the CB level in g-C_3_N_4_-SiW_11_.

The hybrid catalysts of g-C_3_N_4_ with POM-derived metal oxides were prepared by the thermal decomposition of the mixture of g-C_3_N_4_ and POM precursors. These POM-derived metal oxides increase the H_2_O_2_ production by enhancing photoinduced generation of electrons. Using such a calcination method, the g-C_3_N_4_-PWO [107] and g-C_3_N_4_-CoWO [108] have been prepared. Take g-C_3_N_4_-PWO as an example (Fig. 5d), it showed a H_2_O_2_ yield of 6.3 μmol in 60 min under visible light but in the absence of organic electron donor, outperforming that of the counterparts (Fig. 5e). This is because the incorporation of PWO into the g-C_3_N_4_ framework results in the negative shift of the CB edge of g-C_3_N_4_. The H_2_O_2_ production was achieved formed a two-step single-electron O_2_ reduction reaction routine, namely from 1e^−^ reduction pathway (from O_2_ to ·O_2_^−^), and sequentially followed by another 1e^−^ reduction pathway (from ·O_2_^−^ to H_2_O_2_) (Fig. 5f).

#### Metal/Non-metal Element Doping

**Fig. 6:**
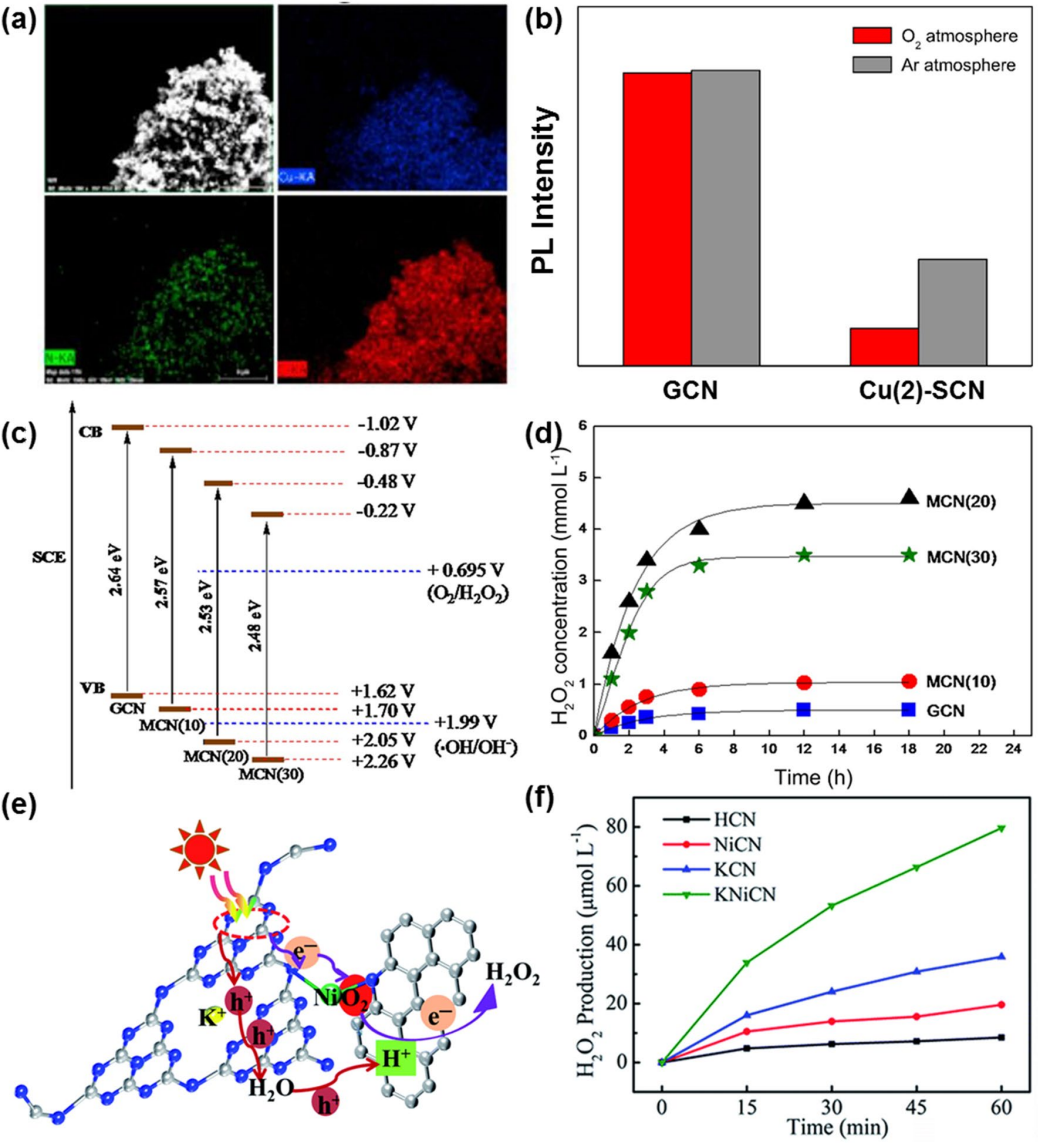
**a** Elemental mapping images of Cu(2)-SCN. **b** PL intensities of GCN and Cu(2)-SCN. Reproduced with permission from Ref. [109]. Copyright 2017 Elsevier. **c** Band gap structures of various catalysts. **d** H_2_O_2_ concentration over catalysts under visible light. Reproduced with permission from Ref. [110]. Copyright 2018 Elsevier. **e** Proposed mechanism of H_2_O_2_ production over the KNiCN catalyst. **f** Photocatalytic H_2_O_2_ production on the catalysts in pure water. Reproduced with permission from Ref. [111]. Copyright 2021 Royal Society of Chemistry

Doping of metal/non-metal element into g-C_3_N_4_ modulates the bandgap of g-C_3_N_4_ and further alters the transfer directions of charge carriers, thereby adjusting electronic, optical and other physical properties of g-C_3_N_4_. Moreover, the heteroelements on g-C_3_N_4_ change the reaction pathways of the H_2_O_2_ generation. Therefore, doping of metal/non-metal element into g-C_3_N_4_ has been proposed as a potential strategy to improve the performance of photocatalytic H_2_O_2_ generation. In a typical case, Cu-doped g-C_3_N_4_ microspheres, denoted as Cu-SCN, have been synthesized using a modified template method (Fig. 6a) [109]. The Cu species were inserted into the interstitial position of SCN with a special mesoporous structure, resulting in the formation of the coordinated Cu–N bonds. The Cu-doping not only decreased the bandgap of g-C_3_N_4_ to enhance its capture of visible light, but also retarded the recombination of photogenerated carriers, as testified from lower PL intensity of Cu(2)-SCN than that of GCN. In addition, Cu(2)-SCN showed a weaker PL intensity in O_2_ atmosphere when compared with that in Ar (Fig. 6b). The performance of photocatalytic H_2_O_2_ production using the Cu-doped g-C_3_N_4_ photocatalyst was thus superior to that of the g-C_3_N_4_ photocatalyst_._ From the X-ray photoelectron spectroscopy (XPS) of Cu-doped g-C_3_N_4_ photocatalyst, Cu^+^ was observed in its XPS spectrum of Cu 2*p*. Such Cu(I)-N site thus served as the adsorption center for molecular O_2_ to increase the O_2_ adsorption. Moreover, it changes the transfer pathways of photoelectrons from the catalyst to the adsorbed O_2_, altering in a two-step single-electron reaction pathway to a one-step two-electron reduction process. In the other study, K^+^ and Na^+^ ions have been co-doped into g-C_3_N_4_ through a molten salt method [110]. The incorporation of alkali metals in g-C_3_N_4_ tuned the band gap of g-C_3_N_4_, increased its specific surface area and reduced the size of g-C_3_N_4_ layered structure. Therefore, this alkali metals co-doped g-C_3_N_4_ photocatalyst can enhance the visible-light adsorption and promote the separation of photoelectron-hole pairs to boost photocatalytic performance toward H_2_O_2_ production. In more detail, the VB potential of GCN(20) is +2.05 V, more positive than that (+1.99 V) of ^·^OH/OH^−^. This was derived from alkali metal incorporation. In this context, the reaction process of photocatalytic H_2_O_2_ generation was changed from a single pathway (O_2_ → H_2_O_2_) to a dual channel pathway (O_2_ → H_2_O_2_ and OH^−^ → ^·^OH → H_2_O_2_), a very attractive method for practical solar-to-chemical applications (Fig. 6c). The MCN(20) produced the H_2_O_2_ concentration of 4.6 mmol L^−1^, more than 9 times higher than that produced by GCN (Fig. 6d). Furthermore, a crystalline g-C_3_N_4_ modified with K^+^, Ni and N-doped carbon (KNiCN) was fabricated to overcome the inherent structural defects and low carrier separation efficiency [111]. The synergistic effects between K^+^, Ni species and N-doped carbon increased the light adsorption, improved charge separation and boosted O_2_ adsorption and selectivity for the H_2_O_2_ generation. A two-electron reduction pathway was suggested (Fig. 6e). This photocatalyst displayed a photocatalytic H_2_O_2_ yield of as high as 79.6 μM in O_2_-saturated pure water (Fig. 6f).

In addition to metal doping of g-C_3_N_4_, halogen doping, another classic non-metal doping has been used to synthesize photocatalysts for photocatalytic H_2_O_2_ production. In these cases, the incorporation of halogen into g-C_3_N_4_ not only reduces the bandgap (to enhance the visible-light absorption and improve the electrical conductivity), but also boost charge carrier transmission rates (without damaging of main structures of the g-C_3_N_4_ polymer). These features benefit much photocatalytic H_2_O_2_ synthesis [112, 113]. For example, halogen doped g-C_3_N_4_, prepared by means of a hydrothermal method in saturated NH_4_X (X = Cl, Br) solution followed with a post-treatment, has more negative CB potential than g-C_3_N_4_ [114]. This catalyst is thus beneficial for the O_2_ reduction into H_2_O_2_. For example, a Br-doped g-C_3_N_4_ photocatalyst showed higher photocatalytic H_2_O_2_ production capacity (1.99 mmol L^−1^) in comparison with the counterparts. The incorporation of multiple heteroelements into the g-C_3_N_4_ framework with various configurations was also demonstrated to tune the bandgap of g-C_3_N_4_ [115]. One co-doped g-C_3_N_4_ photocatalyst exhibited an outstanding photocatalytic activity for the H_2_O_2_ generation, which is 17–25 times higher than that of bare g-C_3_N_4_.

### TiO_2_ Photocatalysts

TiO_2_ is the most famous photocatalyst since the discovery of its photocatalytic activity by Fujishima and Honda for the first time in 1972 [128]. It possesses many advantages for photocatalytic reactions, such as appropriate site for the CB, good physical and electrical properties, stable chemical structures and outstanding biocompatibility [129]. The potential of the CB of TiO_2_ ($E^{\theta }$) is − 0.5 V_NHE_, which is negative enough to drive a 2e^−^ ORR for the H_2_O_2_ production [16]. The generated photoelectrons in their CBs reduce O_2_ to form H_2_O_2_, following the reaction of O_2_ + 2 H^+^  + 2 e^−^  → H_2_O_2_. While these simultaneously generated holes in their VBs oxidize water to generate O_2_ and H^+^, following the process of H_2_O + 2 h^+^  → 1/2 O_2_ + 2 H^+^. The free Gibbs Energy (Δ*G*) of such a photocatalytic process is 117 kJ mol^−1^. An uphill reaction indicates thermodynamical unfavorablity [13, 120]. TiO_2_ photocatalysts also have been confirmed to feature low cost, low toxicity, high chemical- and photo-stability during the H_2_O_2_ production [130–132].

However, the concentration of H_2_O_2_ produced by pure TiO_2_ photocatalyst has been only confined to the micromolar level (< 0.2 mM) [133]. This is because TiO_2_ photocatalyst can be excited only by UV light irradiation to generate photocarriers, due to the large band gap of TiO_2_ photocatalyst [47]. Besides, the formed H_2_O_2_ on the TiO_2_ surface is not stable enough and is easy to be converted to Ti-OOH complexes when it is in touch with the Ti–OH groups on the TiO_2_ surface. Subsequently, Ti-OOH is decomposed, following the reduction reaction of Ti-OOH + H^+^  + e^−^ → Ti–OH + OH^−^ [134]. In order to address the drawbacks of these TiO_2_ photocatalysts, surface modification strategies on TiO_2_ photocatalysts have been validated and applied to facilitate the photocatalytic H_2_O_2_ production, covering loading precious metal nanoparticles, modifying graphene quantum dots and complexing with cation and anion surfaces (Table 2).

**Table 2:** Summary of TiO_2_ photocatalysts for the H_2_O_2_ production

Catalyst	Organic sacrificial agent	Irradiation condition	H_2_O_2_ yield	AQY (%)	Refs.
Co@TiO_2_	–	*λ* = 400 nm	1.71 mmol dm^−3^(60 min)	–	[131]
TiO_2_	Benzylic alcohol	*λ* > 280 nm	40 mM (12 h)	29.1 (*λ* = 334 nm)	[148]
Au/SnO_2_–TiO_2_	Alcohol	UV	~ 15 mM (25 h)	–	[149]
Au/[SnO_2_–NR#TiO_2_]	Ethanol	*λ* > 430 nm	~ 60 μM (6 h)	–	[150]
RuO_2_#TiO_2_-Au	–	*λ* > 300 nm	~ 80 μM (1 h)	–	[151]
Au/TiO_2_	C_2_H_5_OH	*λ* > 300 nm	~ 6 mM (24 h)	13 (*λ* = 355 ± 23 nm)	[133]
BM-Au/TiO_2_ − CO_3_^2−^	Formic acid	*λ* > 430 nm	640 ± 60 μM(1 h)	5.4 (*λ* = 530 nm)	[135]
AuAg/TiO_2_	Ethanol	*λ* > 280 nm	3.4 mM (12 h)	–	[139]
Pd/APTMS/TiO_2_	–	simulated sunlight irradiation	150 μM h^−1^	–	[152]
SN-GQD/TiO_2_	2-propanol	*λ* ≥ 300 nm	451 μM (60 min)	–	[143]
Nf-SNG/TiO_2_	2-propanol	*λ* ≥ 300 nm	745.5 μM (120 min)	–	[144]
HTNT-CD	–	*λ* > 365 nm	84.7 μmol (1 h)	5.2 (*λ* = 365 nm)	[153]
Cu^2+^/TiO_2_	–	*λ* = 300–400 nm	8 μM (5 min)	–	[145]
F-TiO_2_	HCOOH	*λ* = 360 nm	1–1.3 mM	–	[147]
Zn^2+^/TiO_2_	–	mercury lamp (125 W)	0.22 mM (3 h)	–	[146]
TiO_2_-PW_9_	Benzyl alcohol	*λ* = 200–1100 nm	38.2 μmol (2 h)	–	[154]
rGO/TiO_2_/CoPi	2-propanol	*λ* ≥ 320 nm	80 μM (3 h)	–	[155]

#### Loading Precious Metal Nanoparticles

**Fig. 7:**
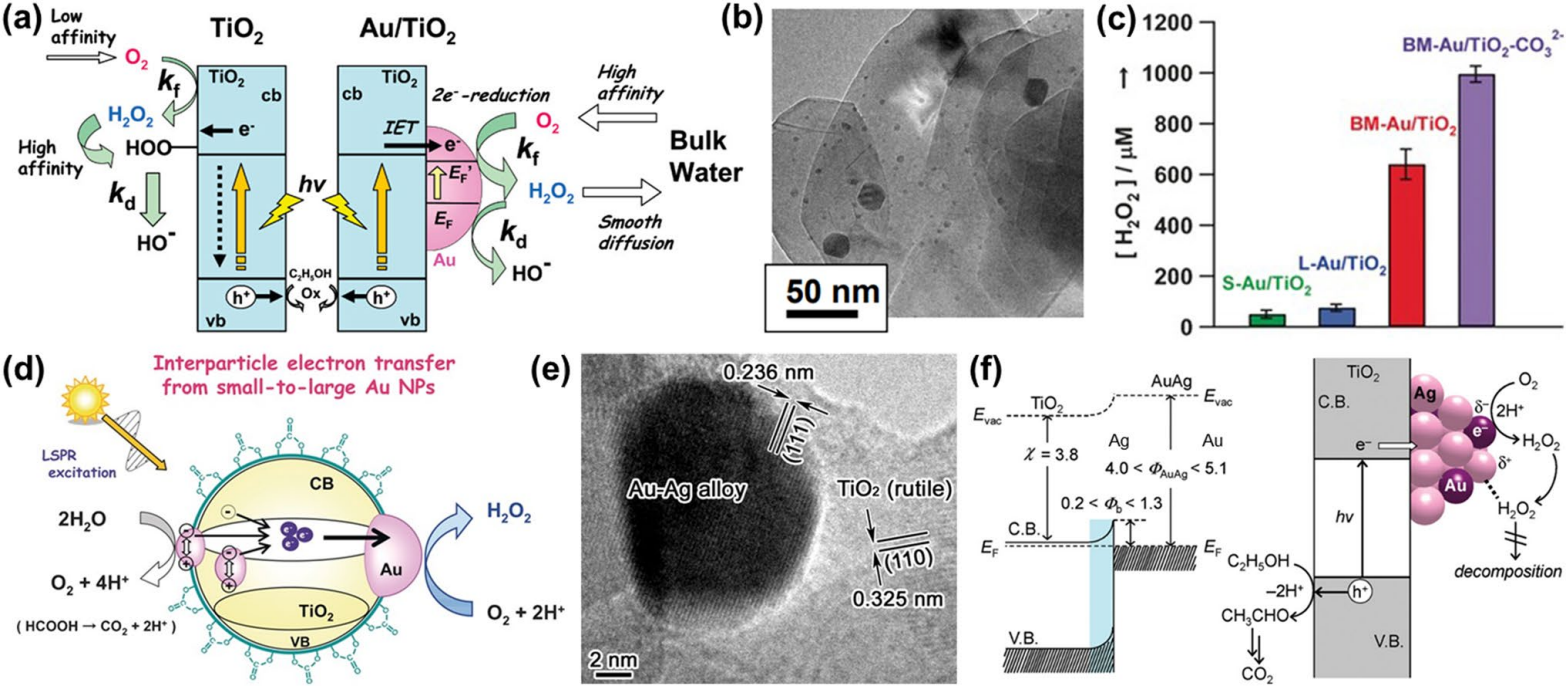
**a** Mechanism of the H_2_O_2_ formation over the Au/TiO_2_ photocatalyst. Reproduced with permission from Ref. [133]. Copyright 2010 American Chemical Society. **b** TEM image of the BM-Au/TiO_2_ photocatalyst. **c** Photocatalytic H_2_O_2_ production on various photocatalysts. **d** Proposed mechanism of the BM-Au/TiO_2_-CO_3_^2−^ plasmonic catalyst in the photocatalytic H_2_O_2_ generation. Reproduced with permission from Ref. [135]. Copyright 2016 Wiley–VCH Verlag GmbH & Co. KGaA, Weinheim. **e** HRTEM image of the Au_0.1_Ag_0.4_/TiO_2_ photocatalyst. **f** Schematic energy-band and mechanism of the H_2_O_2_ production on the AuAg/TiO_2_ photocatalyst. Reproduced with permission from Ref. [139]. Copyright 2012 American Chemical Society

The NPs from individual precious metals have been loaded on the TiO_2_ surface to enhance the photocatalytic activity of TiO_2_ photocatalysts. In 2010, the synthesis of the Au/TiO_2_ photocatalyst has been reported using a deposition–precipitation (DP) method [133]. The as-obtained Au/TiO_2_ photocatalyst exhibited a high yield in the photocatalytic H_2_O_2_ production. The reported H_2_O_2_ concentration reached 10 mM under UV-irradiation. The mechanism of the H_2_O_2_ formation on this Au/TiO_2_ photocatalyst was explained as following (Fig. 7a). The oxidation and reduction sites on the Au/TiO_2_ photocatalyst are separated to TiO_2_ and Au NPs, respectively. The activity for photocatalytic H_2_O_2_ formation can be adjusted by *k*_f_ (the formation rate constant) and *k*_d_ (the decomposition rate constant). In another work, Au NPs with a bimodal size distribution were loaded on rutile TiO_2_ (BM-Au/TiO_2_), which was further modified with carbonates to obtain BM-Au/TiO_2_-CO_3_^2−^ (Fig. 7b) [135]. The UV/Vis absorption spectra of BM-Au/TiO_2_-CO_3_^2−^ indicated that the loading of Au NPs results in strong and extensive absorption in visible light region because of the LSPR of Au NPs. This BM-Au/TiO_2_ photocatalyst yielded 640 ± 60 μM H_2_O_2_ under visible-light irradiation for 1 h when 4% HCOOH was presented as the sacrificial agent. Such a yield was much higher than that of single small Au/TiO_2_ photocatalyst (50 μM) and single large Au/TiO_2_ photocatalyst (75 μM) (Fig. 7c). The improvement on the H_2_O_2_ yield on the BM-Au/TiO_2_-CO_3_^2−^ was believed to be mainly originated from the occurred long-range charge separation by the visible-light-induced, vectorial interfacial electron transfer in the way of S–Au → CB-(TiO_2_) → L-Au. It is known that the generated H_2_O_2_ on a pure TiO_2_ photocatalyst is easily degraded since it can be reduced by the peroxide species (Ti_s_-OOH) on the TiO_2_ surface. However, the modification of CO_3_^2−^ onto the TiO_2_ surface efficiently inhibits such reductive degradation of H_2_O_2_. The concentration of generated H_2_O_2_ on this BM-Au/TiO_2_-CO_3_^2−^ thus reached about 1 mM. The mechanism of the H_2_O_2_ generation on the BM-Au/TiO_2_-CO_3_^2−^ photocatalyst includes plasmonic effect and photocatalytic O_2_ reduction (Fig. 7d**)**. It has been further reported that the photocatalytic kinetics for the H_2_O_2_ formation is dependent on temperature and pH value for the deposition of Au NPs on anatase TiO_2_ when a heating temperature-varied deposition–precipitation technique was applied [136]. The amount of generated H_2_O_2_ under the irradiation was increased with a decrease of the temperature and pH in the reaction system. The highest H_2_O_2_ concentration produced on the Au/anatase TiO_2_ photocatalyst was about 17 mM at the temperature of 5 °C in the solution with a pH value of 2. In conclusion, these introduced metal Au NPs on the TiO_2_ surface prohibit the H_2_O_2_ decomposition that is induced by the Ti–OH species. Meanwhile, they capture photogenerated electrons in the CB of TiO_2_, leading to retarded recombination of photogenerated carriers. The output of the H_2_O_2_ production on these photocatalysts was obviously increased. The H_2_O_2_ concentration even reached the millimolar level. Nevertheless, the generated H_2_O_2_ molecules are inclined to be adsorbed on Au NPs due to their strong interactions. Subsequently, spontaneous decomposition of H_2_O_2_ occurs: H_2_O_2_ + e^−^ → ^·^OH + OH^−^ [137, 138].

To promote the H_2_O_2_ formation and simultaneously inhibit its decomposition on the TiO_2_ photocatalysts, the NPs from bimetal alloys have been deposited on the TiO_2_ surface to enhance the photocatalytic activity. For example, a bimetal alloy catalyst (AuAg/TiO_2_) has been designed and applied for photocatalytic H_2_O_2_ generation (Fig. 7e) [139]. The H_2_O_2_ concentration produced by the Au_0.1_Ag_0.4_/TiO_2_ photocatalyst was as high as 3.4 mM, approximately 7 times higher than that produced on a pure TiO_2_ photocatalyst. This excellent photocatalytic performance has been attributed to the following two aspects (Fig. 7f). Firstly, the work function of the alloy AuAg is located between Au and Ag. The alloy/TiO_2_ photocatalyst produces a barrier, of which energy is larger than that of the Ag/TiO_2_ photocatalyst but smaller than that of Au/TiO_2_ photocatalyst. Effective separation of hole-electron pairs is thus possible. Secondly, electron transfer from Ag to Au takes place, because Au atoms have a higher electronegativity than Ag atoms. In other words, the electron density of Au is increased, suppressing the H_2_O_2_ adsorption on Au atoms. Such statements were further proved using the DFT simulations. The affinity between the H_2_O_2_ and Au atoms was confirmed to be weakened via the alloy effect. It was taken place that H_2_O_2_ is inclined to be adsorbed on Ag atoms, thus inhibiting the H_2_O_2_ decomposition. In another case, the midgap state in the Ag_2_Au_2_@TiO_2_ (101) photocatalyst was reveal to own a suitable position for the H_2_O_2_ production via a photocatalytic reaction, as demonstrated by the DFT analysis. More importantly, the photoresponse of these bimetal alloy photocatalytic systems is rapidly promoted in visible and infrared light region, improving the H_2_O_2_ yield [140].

#### Modifying Graphene Quantum Dots

**Fig. 8:**
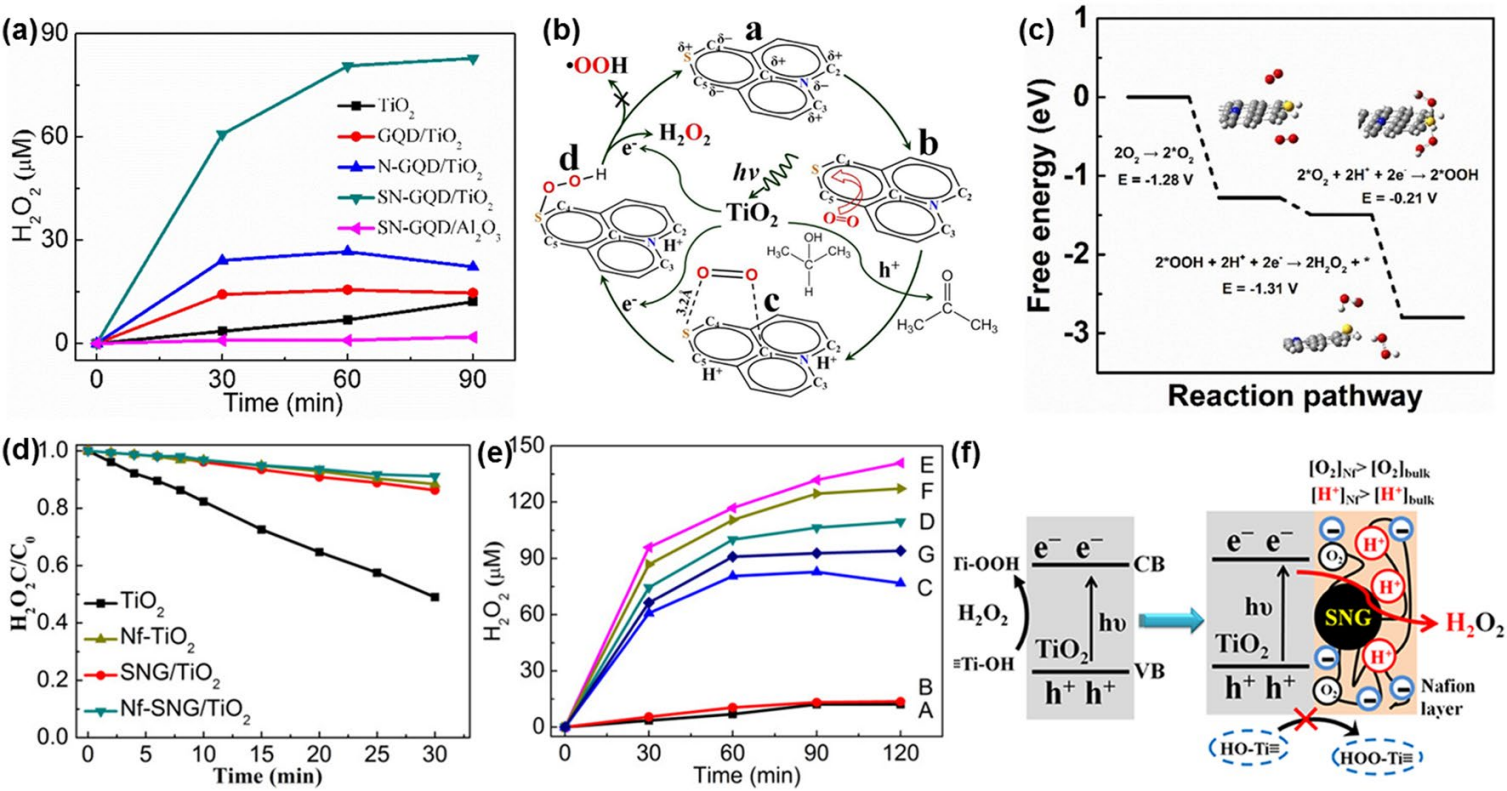
**a** Photocatalytic H_2_O_2_ generation on the catalysts. **b** Proposed mechanism of photocatalytic H_2_O_2_ formation over the SN-GQD/TiO_2_ photocatalyst. **c** Free energy diagrams of reaction pathways. Reproduced with permission from Ref. [143]. Copyright 2018 Elsevier. **d** Photocatalytic decomposition and **e** production of H_2_O_2_ on different catalysts under visible light. **f** Photocatalytic reaction mechanism for H_2_O_2_ formation on the Nf-SNG/TiO_2_ photocatalyst. Reproduced with permission from Ref. [144]. Copyright 2019 American Chemical Society

Graphene quantum dots (GQDs) feature advantages of high chemical stability, good biocompatibility, large surface area and high extinction efficiencies (that are derived from the atomically thin *sp*^2^ carbon structure of graphene). GQDs exhibit unique luminescent characteristic. Therefore, GQDs have attracted extensive attention in various photocatalytic applications. Especially, the sensitization of GQDs with other wide band gap photocatalysts (e.g., TiO_2_) can extend light-adsorption range from ultraviolet to the visible region [141, 142]. For example, sulfur and nitrogen co-doped graphene quantum dots (SN-GQDs) have been combined with the TiO_2_ photocatalyst (SN-GQD/TiO_2_) and further applied for photocatalytic H_2_O_2_ production [143]. As confirmed from related UV–vis diffuse reflectance spectra (DRS), SN-GQD/TiO_2_ exhibited obviously enhanced adsorption in visible light region when compared with the pure TiO_2_ photocatalyst. On its surface, more than 82.8 μM H_2_O_2_ was produced under visible light irradiation only for 90 min. Such a yield was about 5.3 and 3.1 times larger than that generated on the GQD/TiO_2_ photocatalyst and the N-GQD/TiO_2_ suspensions under the same conditions, respectively (Fig. 8a). Theoretical calculation and free energy diagram analysis showed that the H_2_O_2_ generation on the SN-GQD/TiO_2_ photocatalyst followed a proton-coupled electron transfer (PCET) mechanism. Namely, high selectivity of the H_2_O_2_ generation on the SN-GQD/TiO_2_ photocatalyst was realized via a 2e^−^ ORR pathway (Fig. 8b, c). A Nafion layer was further introduced into the SN-GQD/TiO_2_ (Nf-SNG/TiO_2_) to facilitate the H_2_O_2_ generation [144]. The GQDs coupled with a Nafion layer were found not only to improve the visible light adsorption, but also to significantly hinder the H_2_O_2_ decomposition (Fig. 8d). The yield of photocatalytic H_2_O_2_ production on the Nf-SNG/TiO_2_ photocatalyst with 3.5% Nafion content was 141 μM after 120 min visible light irradiation than that obtained on the counterparts (Fig. 8e). Related photocatalytic reaction mechanism of the H_2_O_2_ formation on Nf-SNG/TiO_2_ was testified to follow a two-electron-dominated ORR pathway (Fig. 8f). It was impacted by GQDs sensitization, different from photocatalytic H_2_O_2_ production on the pure TiO_2_ photocatalyst.

#### Complexing with Cation and Anion Surfaces

The surface states are important during photocatalytic H_2_O_2_ generation, since photocatalytic reactions are surface-controlled processes. Therefore, various surface modification approaches have been developed to modulate photocatalytic activities of TiO_2_ photocatalysts. Among them, complexing cations or anions onto the TiO_2_ surface has been wildly accepted as the simplest one to effectively boost the photocatalytic H_2_O_2_ production. For example, the inner spherical surface of the metal cations can modulate interfacial electron transfer via suppressing the surface trapping sites for photogenerated carriers during the photocatalytic process. Taking the modification of TiO_2_ by Cu^2+^ ion as an example, it has been revealed that complexing with a small amount of Cu^2+^ onto the TiO_2_ surface significantly promoted the photocatalytic activity of H_2_O_2_ production [145]. A 20-fold increment in the amount of generated H_2_O_2_ was achieved under UV irradiation in comparison with that on the TiO_2_ photocatalyst without Cu^2+^ modification. When the concentration of Cu^2+^ was in the range of 30–40 μM, the performance for photocatalytic H_2_O_2_ generation remained stable. Such enhanced activity of photocatalytic H_2_O_2_ production was proposed to be dependent on the optimization of the TiO_2_ surface state through Cu^2+^ modification, eventually promoting the H_2_O_2_ formation on the TiO_2_ surface via a 2e^−^ ORR pathway. In another case, Zn^2+^ ions were complexed onto the TiO_2_ photocatalyst [146]. This Zn^2+^ modified TiO_2_ photocatalyst exhibited obviously improved performance of photocatalytic H_2_O_2_ generation. Since Zn^2+^ ions block the ≡Ti–OH sites, the complexation of peroxide/superoxide species on the TiO_2_ surface is thus much limited, resulting in reduced H_2_O_2_ decomposition.

In addition to cations, complexing with anions onto the TiO_2_ photocatalyst has been also applied for the photocatalytic H_2_O_2_ production. For instance, the fluorinated TiO_2_ photocatalyst exhibited a boosted reaction toward photocatalytic H_2_O_2_ generation. Stemming from surface state modulation via F^−^ modification, this fluorinated TiO_2_ photocatalyst suppresses the Ti-OOH formation. It produced H_2_O_2_ with a concentration of 1.3 mM under UV light irradiation, one of the best activities among the reported TiO_2_ photocatalysts for the H_2_O_2_ generation [147].

### BiVO_4_ Photocatalysts

**Fig. 9:**
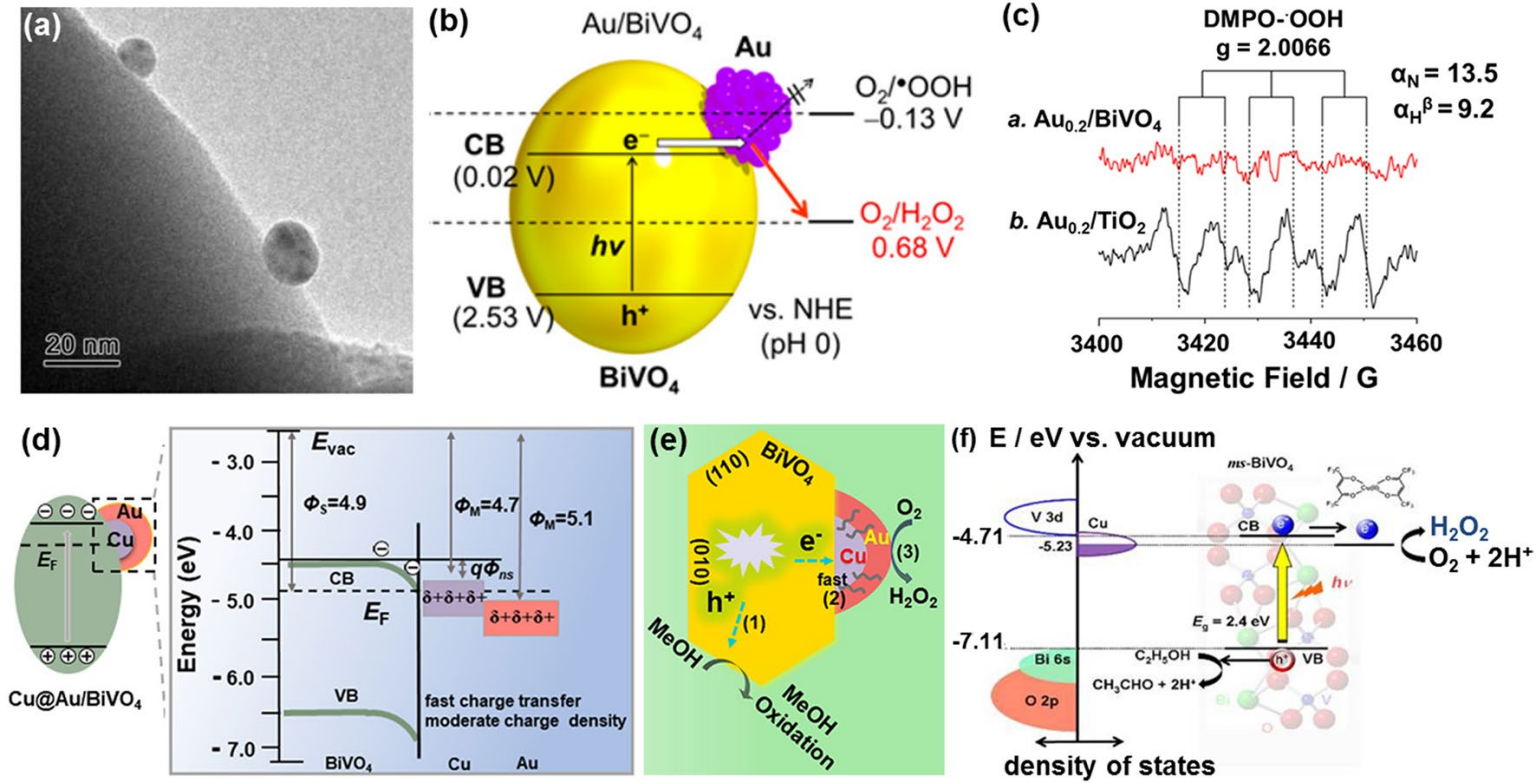
**a** TEM image of an Au_0.2_/BiVO_4_ photocatalyst. **b** Energy diagram of the Au/BiVO_4_ photocatalyst and reduction potential of O_2_. **c** ESR spectra of an Au_0.2_/BiVO_4_ photocatalyst and an Au_0.2_/TiO_2_ photocatalyst in an EtOH/water/O_2_ system with DMPO. Reproduced with permission from Ref. [163]. Copyright 2016 American Chemical Society. **d** Energy diagram of a Cu@Au/BiVO_4_ photocatalyst. **e** Mechanism of H_2_O_2_ generation on a Cu@Au/BiVO_4_ photocatalyst. Reproduced with permission from Ref. [166]. Copyright 2021 American Chemical Society. **f** Energy diagram and the reaction mechanism of a Cu(hfacac)_2_/ms-BiVO_4_ photocatalyst. Reproduced with permission from Ref. [167]. Copyright 2020 American Chemical Society

In addition to TiO_2_ photocatalysts, some more complex inorganic oxides have also been employed for photocatalytic H_2_O_2_ generation. Among them, bismuth vanadate (BiVO_4_) is one classic representative of bimetallic oxide photocatalyst [156–159]. Again, it is well known a pure TiO_2_ photocatalyst has a wide bandgap and thus can be excited only under UV light irradiation, resulting in insufficient activity for photocatalytic H_2_O_2_ generation in sunlight [160, 161]. Distinguished from a TiO_2_ photocatalyst, BiVO_4_ has its appropriate band structure and is thus active in the visible light region. In other words, it can promote the H_2_O_2_ production via a 2e^−^ ORR pathway under the visible-light irradiation [162]. Unfortunately, pure BiVO_4_ is not conducive to the efficient photocatalytic H_2_O_2_ production, due to its lack of active sites for the 2e^−^ ORR. In this context, nano-Au cocatalyst was introduced onto the BiVO_4_ surface. Stemming from the strong interaction between Au and BiVO_4_, the activation of the special *d*-band electrons promoted the selectivity of the 2e^−^ ORR pathway to efficiently generate H_2_O_2_ under visible light irradiation (Fig. 9a) [163]. This is because of the edge in the CB of BiVO_4_ was disclosed to be 0.02 V, more positive than that (−0.13 V) of one-electron reduction of O_2_ and more negative than that (0.68 V) of two-electron reduction of O_2_ (Fig. 9b). The inhibition of single electron reduction over the Au/BiVO_4_ surface was further identified by electron spin resonance (ESR) spectra, where Au_0.2_/BiVO_4_ exhibits almost no signal, while Au_0.2_/TiO_2_ displays distinctive signals (Fig. 9c). The latter was ascribed to the DMPO − ·OOH spin adduct formation.

Nevertheless, the activity of the BiVO_4_ photocatalyst toward photocatalytic H_2_O_2_ production still remains unsatisfactory. This is mainly attributed to the formed built-in field between Au and BiVO_4_, which inhibits the transfer of photogenerated electrons as well as accumulates negative charges of Au to deteriorate the 2e^−^ ORR pathway [164, 165]. In order to overcome this impediment, one Cu@Au/BiVO_4_ photocatalyst was designed and obtained using combined photodeposition and galvanic displacement methods [166]. The Cu species was found to facilitate the transfer of photogenerated electrons from BiVO_4_ to Au. The accumulation of negative charges on Au was then reduced, resulting in the enhanced activity for the photocatalytic H_2_O_2_ production (Fig. 9d, e). Specifically, the ohmic contact was expected to be produced between Cu and BiVO_4_ since their work functions (*Φ*) are different: ${\Phi }_{Cu}$= 4.7 eV and ${\Phi }_{{BiVO}_{4}}$= 4.9 eV. Transferring of the photogenerated electrons is thus beneficial. The photogenerated electrons that are transferred from Cu to Au eventually boost the two-electron O_2_ reduction pathway, namely the H_2_O_2_ formation. On the other hand, photogenerated charges have been accumulated on the nano-Au cocatalyst in the Cu@Au/BiVO_4_ photocatalyst, leading to the generation of stronger adsorption of O_2_ and HOO^*^ on the Au surface of Cu@Au/BiVO_4_. Consequently, O_2_ reduction to form H_2_O_2_ is accelerated. For a bis(hexafluoroacetylacetonato) Cu(II) adsorbed monoclinic scheelite (ms)-BiVO_4_ photocatalyst (Cu-(hfacac)_2_/ms-BiVO_4_), it exhibited an outstanding activity of photocatalytic H_2_O_2_ generation [167]. An external quantum yield of 0.47% was reported under visible light irradiation (λ_ex_ = 470 nm). Such performance was attributed to the enhanced charge separation by the interfacial electron transfer from ms-BiVO_4_ to the surface complex and the O_2_-enriching effect near the surface of ms-BiVO_4_, as well as outstanding electrocatalysis for a 2e^−^ ORR pathway (Fig. 9f**)**.

### CdS Photocatalysts

CdS has exhibited promising photocatalytic activities in various reactions under visible light irradiation, since the potential of its VB is positive enough to drive water oxidation and meanwhile the potential of its CB is more negative to promote O_2_ reduction. CdS thus has great potential to be applied as a catalyst for photocatalytic H_2_O_2_ production [168–170]. Unfortunately, CdS exhibits relatively low photocatalytic capability toward H_2_O_2_ generation, originating from its weak adsorption capacity for reactants and its poor photostability. More seriously, its easy aggregation can cause the severe recombination of photogenerated carriers [171].

**Fig. 10:**
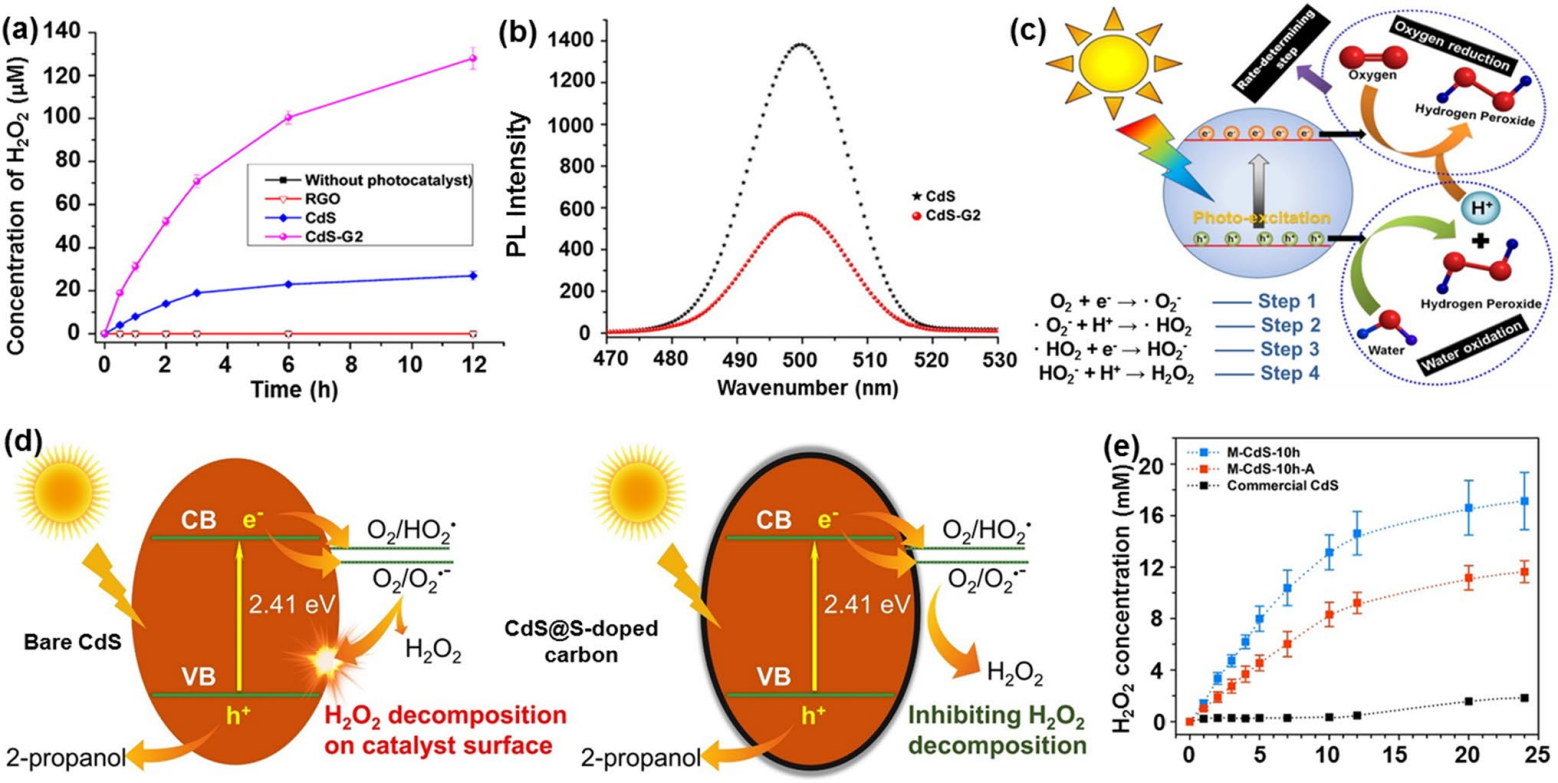
**a** H_2_O_2_ concentration produced on different catalysts. **b** PL spectra of the CdS and CdS-G2 photocatalysts. **c** Mechanism for H_2_O_2_ production on the CdS-G hybrid photocatalyst under sunlight illumination. Reproduced with permission from Ref. [172]. Copyright 2016 Elsevier. Schematic illustration of H_2_O_2_ formation over a CdS photocatalyst and a CdS@S-doped carbon photocatalyst. **e** H_2_O_2_ concentration for 24 h with 20% of 2-propanol. Reproduced with permission from Ref. [174]. Copyright 2020 Elsevier

To improve the performance of CdS for photocatalytic H_2_O_2_ production, many efforts have been made and several strategies have been proposed. One strategy is to complex organic polymers onto CdS. For example, the hybrids of CdS and reduced graphene oxide (CdS-RGO) prepared through a hydrothermal process significantly boosted the kinetics of photocatalytic H_2_O_2_ generation when compared with pure CdS photocatalyst (Fig. 10a) [172]. The CdS-RGO photocatalyst with a 20 wt% RGO content (CdS-G2) produced the H_2_O_2_ concentration of as high as 128 μM under sunlight irradiation for 12 h. Such enhancement was mainly attributed to accelerated separation of photogenerated carriers due to the enhanced electron transfer from the photoexcited CdS to RGO, the increment of visible light absorption and more active reaction sites **(**as demonstrated by their PL spectra (Fig. 10b**))**. The mechanism of photocatalytic H_2_O_2_ production on the CdS-RGO photocatalyst was also discussed (Fig. 10c**)**. In the first step, electron–hole pairs are generated on the CdS-RGO photocatalyst upon its excitation by light irradiation. Water molecules are then oxidized by photogenerated holes, leading to the production of H_2_O_2_ and protons. Simultaneously, O_2_ is reduced to form H_2_O_2_, following a 2e^−^ ORR pathway (step 1–4). These photogenerated electrons have been demonstrated to play a vital role in the process of photocatalytic H_2_O_2_ generation. The H_2_O_2_ yield from a 2e^−^ ORR pathway is the decisive factor when compared with that from the 2e^−^ WOR pathway in the whole photocatalytic process.

Noble metals have also been complexed onto CdS to improve the photocatalytic performance of CdS catalysts. For example, the CdS-Pt and CdS-Au nanorods exhibited enhanced photocatalytic activity to produce H_2_O_2_ when compared with a pure CdS photocatalyst [173]. The Au tip with a smaller size was found to be more efficient as reactive site to form H_2_O_2_ than a Pt tip. This was attributed to different surface reactivity and selectivity related to the metal tip composition. On these CdS-metal photocatalysts, the H_2_O_2_ production through ORR pathway has been confirmed, instead of the WOR pathway. A CdS/sulfur-doped carbon nanocomposite, synthesized by thermal annealing of Cd(EDDA) MOF, exhibited outstanding activity toward photocatalytic H_2_O_2_ production [174]. This is due to the presence of sulfur-doped carbon, which can effectively hinder the H_2_O_2_ decomposition, as experimentally and theoretically testified (Fig. 10d). The H_2_O_2_ concentration generated on a nanocomposite based photocatalyst (M-CdS-10 h) reached 17.1 mM under visible light irradiation when 2-propanol was used as a sacrificial agent (Fig. 10e).

### Metal–organic Frameworks Photocatalysts

**Fig. 11:**
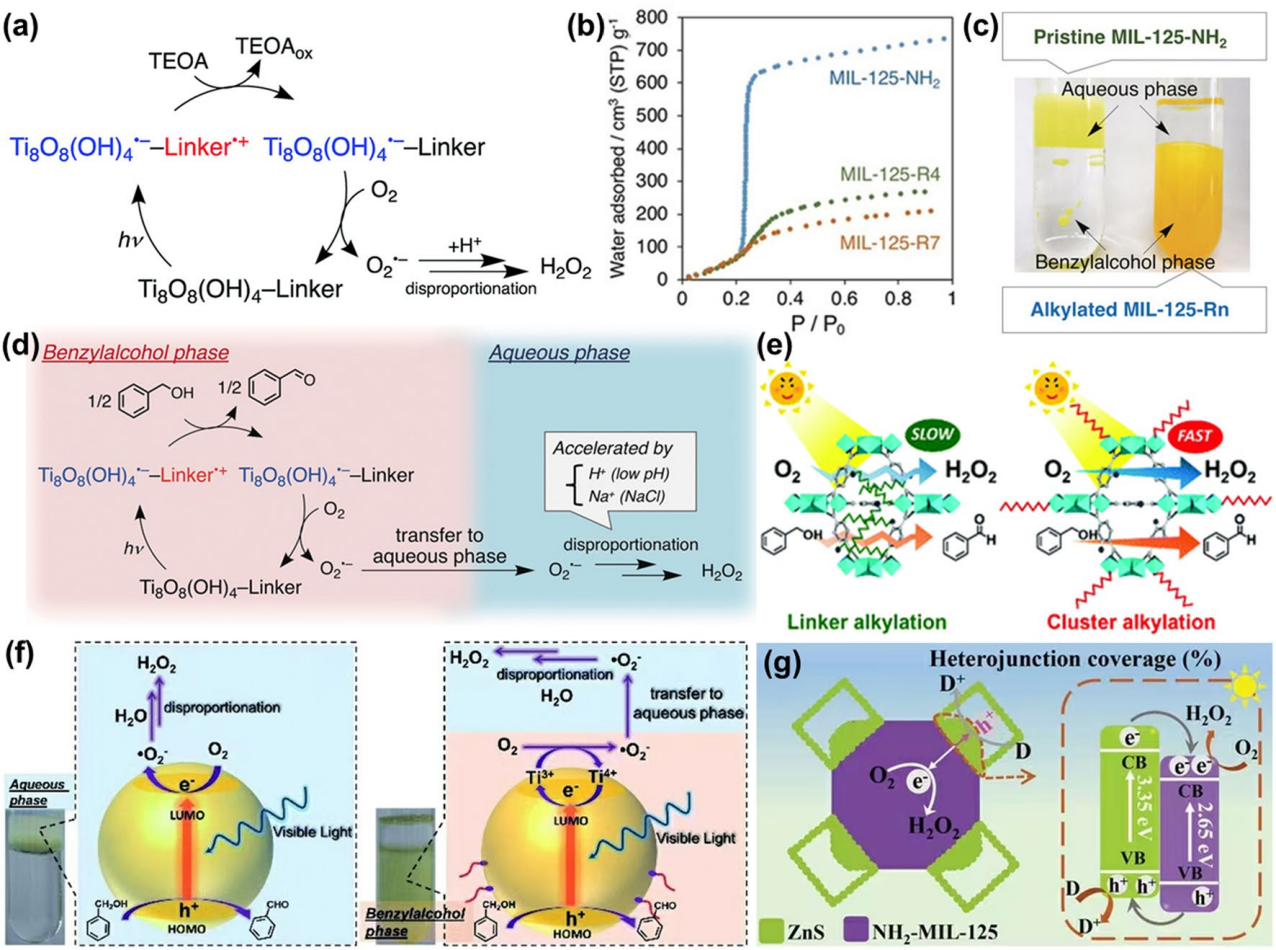
**a** Photocatalytic mechanism of H_2_O_2_ formation on the MIL-125-NH_2_ photocatalyst. Reproduced with permission from Ref. [180]. Copyright 2018 ROYAL SOCIETY OF CHEMISTRY. **b** Water adsorption isotherms for various catalysts. **c** Digital photographs utilizing the two-phase system. **d** Mechanism illustration of H_2_O_2_ generation by the two-phase system. Reproduced with permission from Ref. [181]. Copyright 2019 Wiley–VCH Verlag GmbH & Co. KGaA, Weinheim. **e** Photocatalytic processes of MIL-125-R7 and OPA/MIL-125-NH_2_ photocatalysts. Reproduced with permission from Ref. [182]. Copyright 2019 ROYAL SOCIETY OF CHEMISTRY. **f** Mechanism of photocatalytic H_2_O_2_ production using the Zr_100_-MOF and OPA/Zr_92.5_Ti_7.5_-MOF photocatalyst. Reproduced with permission from Ref. [183]. Copyright 2020 ROYAL SOCIETY OF CHEMISTRY. **g** Photocatalytic O_2_ reduction to H_2_O_2_ over a NH_2_-MIL-125@ZnS heterojunction. Reproduced with permission from Ref. [187]. Copyright 2021 Wiley–VCH GmbH

Metal–organic frameworks (MOFs) own unique porous structures and strong metal–ligand interactions [175]. Similar to semiconductors, MOFs can produce electron–hole pairs upon light irradiation. They thus exhibit great potential in photocatalysis [176–179]. The Ni/MIL-125-NH_2_, obtained via depositing NiO on the MIL-125-NH_2_ was the first MOF photocatalyst used in the photocatalytic H_2_O_2_ synthesis [180]. The mechanism of the H_2_O_2_ production on the Ni/MIL-125-NH_2_ (Fig. 11a) was described as following. Nano NiO effectively inhibits the H_2_O_2_ decomposition through a 2e^−^ ORR pathway under visible light irradiation when benzylalcohol is accompanied as an electron donor. Specifically, MIL-125-NH_2_ produces Ti_8_O_8_(OH)_4_^·−^ and a hole upon its excitation by light irradiation. Once the hole is trapped by triethanolamine (TEOA), O_2_ is reduced to O_2_^·−^ by Ti_8_O_8_(OH)_4_^·−^, followed by rapid H_2_O_2_ formation via the O_2_^·−^ disproportionation reaction in the presence of NiO. Unfortunately, in this case the product was a mixture of H_2_O_2_ and benzaldehyde dissolved in acetonitrile. In other words, further energy-consumption for product separation and purification is required. Later, a two-phase system containing benzylalcohol/water (BA/water), was employed to separate these products [181]. The hydrophilic MIL-125-NH_2_ was conversed to hydrophobic MIL-125-Rn (n = 1, 4 and 7) through the growth of the alkyl chains on the catalyst surface, as clarified using water adsorption isotherms and water contact angles measurements (Fig. 11b). A pristine MIL-125-NH_2_ was proved to exist in the water phase during the photocatalytic H_2_O_2_ process, while alkylated MIL-125-Rn is located in the BA phase (Fig. 11c). During the photocatalytic H_2_O_2_ process (Fig. 11d**)**, O_2_ is firstly reduced to O_2_^·−^ on the hydrophobic MOFs in the BA phase. Subsequently, O_2_^·−^ is transferred to the water phase where H_2_O_2_ is rapidly produced through a disproportionation reaction in the presence of H^+^ or Na^+^. Although this two-phase system inhibited the further reaction of MOFs with H_2_O_2_, its photocatalytic activity of the two-phase system was not satisfying. This is because the grafting of the alkyl chains blocks the pores of the MIL-125-R7, thus greatly reducing its photocatalytic activity. In this regard, a hydrophobic MOF, namely OPA/MIL-125-NH_2,_ was developed. In this case, MIL-125-NH_2_ retained most of its pores using octadecylphosphonic acid (OPA) treatment [182]. Its surface area was 1242 m^2^ g^−1^, comparable to that (1500 m^2^ g^−1^) of MIL-125-NH_2_. Under visible light irradiation for 3 h, the concentration of generated H_2_O_2_ on this OPA/MIL-125-NH_2_ photocatalyst was approximately 3 times larger than that of MIL-125-R7. The enhanced activity was attributed to the rapid diffusion of O_2_^·−^ through the unblocked pores of the OPA/MIL-125-NH_2_ photocatalyst, which prevents the H_2_O_2_ decomposition (Fig. 11e). A hydrophobic titanium doped zirconium-based MOF (OPA/Zr_100-x_Ti_x_-MOF) also exhibited a high rate of H_2_O_2_ production (9.7 mmol L^−1^ h^−1^), where the Ti species played a role in effectively promoting electron transfer from photoexcited linkers of the MOF to Ti, inhibiting the recombination of photogenerated electron–hole pairs in the hydrophobic MOF matrix (Fig. 11f**)** [183].

The construction of the heterojunction by combining MOFs with other semiconductors also effectively strengthens H_2_O_2_ production since the separation of photogenerated electron–hole pairs can be promoted [184–186]. For example, on the MOF@MS heterostructure (NH_2_-MIL-125@ZnS) the photocatalytic synthesis of H_2_O_2_ was reported via a 2e^−^ ORR pathway (Fig. 11g) [187]. This heterostructure presents a spatially separated architecture, where ZnS nanocages are selectively adhered on the four corners of a cake-like NH_2_-MIL-125 body. The coverage of heterojunction was controlled by altering the diameter of ZIF-8/ZnS. In this way, the regulation of the absorption of light, the generation of electron–hole pairs, the charge separation and accessibility were doable, finally leading to adjusted performance of photocatalytic H_2_O_2_ production. During the photocatalytic process, photogenerated electrons transfer from ZnS to NH_2_-MIL-125, while holes migrate from NH_2_-MIL-125 to ZnS, resulting in excellent performance. When the heterojunction coverage is ≈45.1%, the NH_2_-MIL-125@ZnS photocatalyst displays a H_2_O_2_ yield of as high as 120 mM g^−1^ h^−1^.

## Electrocatalytic H_2_O_2_ Synthesis

### Electrocatalytic H_2_O_2_ Synthesis through a 2e^−^ ORR Pathway

2e^−^ ORR has been considered as one of the most vital routes in electrocatalytic H_2_O_2_ synthesis [188, 189]. However, its selectivity is limited by the competition from the 4e^−^ reduction pathway, namely the reduction of O_2_ to generate H_2_O. The fact behind is that O_2_ has a more negative standard potential to form H_2_O than that for the H_2_O_2_ generation [52, 190, 191]. Further electro-reduction of H_2_O_2_ to H_2_O is thus thermodynamically favorable, which severely hinders the yield of H_2_O_2_ from a 2e^−^ ORR pathway. In addition, the spontaneous H_2_O_2_ disproportionation to produce H_2_O and O_2_ prohibits the 2e^−^ ORR pathway, lowering the selectivity of H_2_O_2_ production. Therefore, it is prerequisite to design electrocatalysts to promote the preferential H_2_O_2_ formation as well as to facilitate rapid diffusion of H_2_O_2_ away from the reactive interface. The reported electrocatalysts using the 2e^−^ ORR pathway for the H_2_O_2_ production can be divided into two categories: noble-metal-based and carbon-based electrocatalysts. In the following session, these reported electrocatalysts are systematically classified and their performance toward the H_2_O_2_ is detailed (Table 3).

**Table 3:** Summary of electrocatalytic H_2_O_2_ production via a 2e^−^ ORR pathway

Catalyst	Electrolyte	Onset potential (V_RHE_)	Applied potential (V_RHE_)	Selectivity (%)	*n*	Refs.
*Noble metal-based catalysts*						
C(Pt)/C-3/4 h	1 M HClO_4_	~ 0.7 (− 0.05 mA cm^−2^)	0.1	41	3.2	[200]
Pt-SA/rGO	0.1 M KOH	0.964	0.3–0.8	~ 95	2–2.3	[195]
NC-Ag/NHCS	0.1 M HClO_4_	0.82 (0.1 mA cm^−2^)	0.2–0.7	89–91	~ 2	[249]
Pd^δ+^-OCNT	0.1 M HClO_4_	0.70	0.3–0.7	95–98	–	[201]
Pt/HSC	0.1 M HClO_4_	0.71	0.1–0.7	96	2.1	[197]
Au-Pt-Ni NRs	0.1 M KOH	–	0.45–0.55	95	2.11 (0.5 V)	[250]
*Carbon-based catalysts*						
o-GOMC-1	0.1 M KOH	0.81	–	> 90	–	[218]
HPC-H24	pH 1	–	− 0.1–(− 0.5)	80.9–95.0	2.10–2.38	[215]
MesoC/ MicroC	0.1 M KOH	~ 0.7	–	> 70	~ 2	[217]
G-M1	0.1 M KOH	0.86	0.358	82.07	2.35	[219]
O-CNTs	0.1 M KOH	0.7 (1 mA cm^−2^)	0.4–0.65	~ 90	–	[22]
OCNS_900_	0.1 M KOH	0.825	0.75–0.55	90 (0.7 V)	2.2–2.3	[251]
aCB	0.1 M KOH	0.821 V	0.4–0.7	> 94	–	[222]
CB600	0.1 M Na_2_SO_4_	− 0.15 V_Ag/AgCl_	− 0.35–(− 0.6)	52.6–56.1	–	[223]
rGO_-KOH_	0.1 M KOH	–	–	~ 100	–	[224]
GNP_C=O,1_	0.5 M H_2_SO_4_	0.826 (0.15 mA cm^−2^)	0.75	97.8	~ 2	[225]
OMPC4	0.1 M KOH	–	0.42	87	2.2	[220]
HMCSs	0.1 M KOH	0.82	0.4–0.7	> 95	–	[252]
g-N-CNHs	0.10 M PBS	0.53	0.45	90	2.1	[232]
	0.1 M H_2_SO_4_	0.40	0.3	98	2.4	
	0.1 M NaOH	0.71	0.65	63	3.2	
CG400	0.1 M KOH	0.72 (30 μA cm^−2^)	–	93	~ 2.1	[227]
oxo-G/NH_3_·H_2_O	0.1 M KOH	–	–	> 82	–	[229]
G-COF-950	0.1 M KOH	~ 0.74 (0.1 mA cm^−2^)	− 0.1–0.5	70–75	2.5–2.6	[228]
MNC-50	0.5 M H_2_SO_4_	–	0.1–0.3	> 90	–	[237]
NCMK3IL50_800T	0.5 M H_2_SO_4_	–	0.1–0.3	95–98	2.1	[238]
N, S-MC-1	0.5 M H_2_SO_4_	0.318 V_SHE_	0.06 V_SHE_	76	2.5	[230]
N-doped porous carbon	0.5 M H_2_SO_4_	0.49 (0.01 mA cm^−2^)	0.35	98.5	–	[253]
	0.1 M KOH	0.84		83	–	
HNCS	0.1 M KOH	–	0.7	∼91.9%	–	[254]
HPCS-S	0.1 M KOH	0.77 (0.1 mA cm^−2^)	0.3–0.7	70	2.7	[240]
BN-C1	0.1 M KOH	0.80 (0.5 mA cm^−2^)	–	90	–	[241]
EDTAFeNa-KB-HT1	0.1 M KOH	0.857	0.60–0.75	80–100	2.0–2.4	[242]
Co–N-C	0.5 M H_2_SO_4_	0.83 (0.01 mA cm^−2^)	0.1	80	~ 2.4	[247]
Mn–O/N@NCs-50	0.1 M HClO_4_	–	0.5	87	2.5	[243]
Co_1_-NG(O)	0.1 M KOH	~ 0.8	–	~ 82	–	[248]
Co–N–C	0.1 M HClO_4_	0.74	–	~ 87	–	[244]
Mn–N–C		0.63	–	~ 50	–	
Fe–N–C		0.81	–	~ 30	–	
Co-N_2_-C/HO	0.1 M KOH	0.801 (0.15 mA cm^−2^)	0.7	91.3	2.3	[255]
CDs (Glucose in 1L)	0.1 M KOH	0.68	0.7–0.3	95	2.05	[256]
CMK3-20 s	0.1 M KOH	0.79	0.25–0.75	88–91	–	[212]
	0.1 M K_2_SO_4_	0.46	0.15–0.4	51–75	–	

#### Noble Metal-based Electrocatalysts

**Fig. 12:**
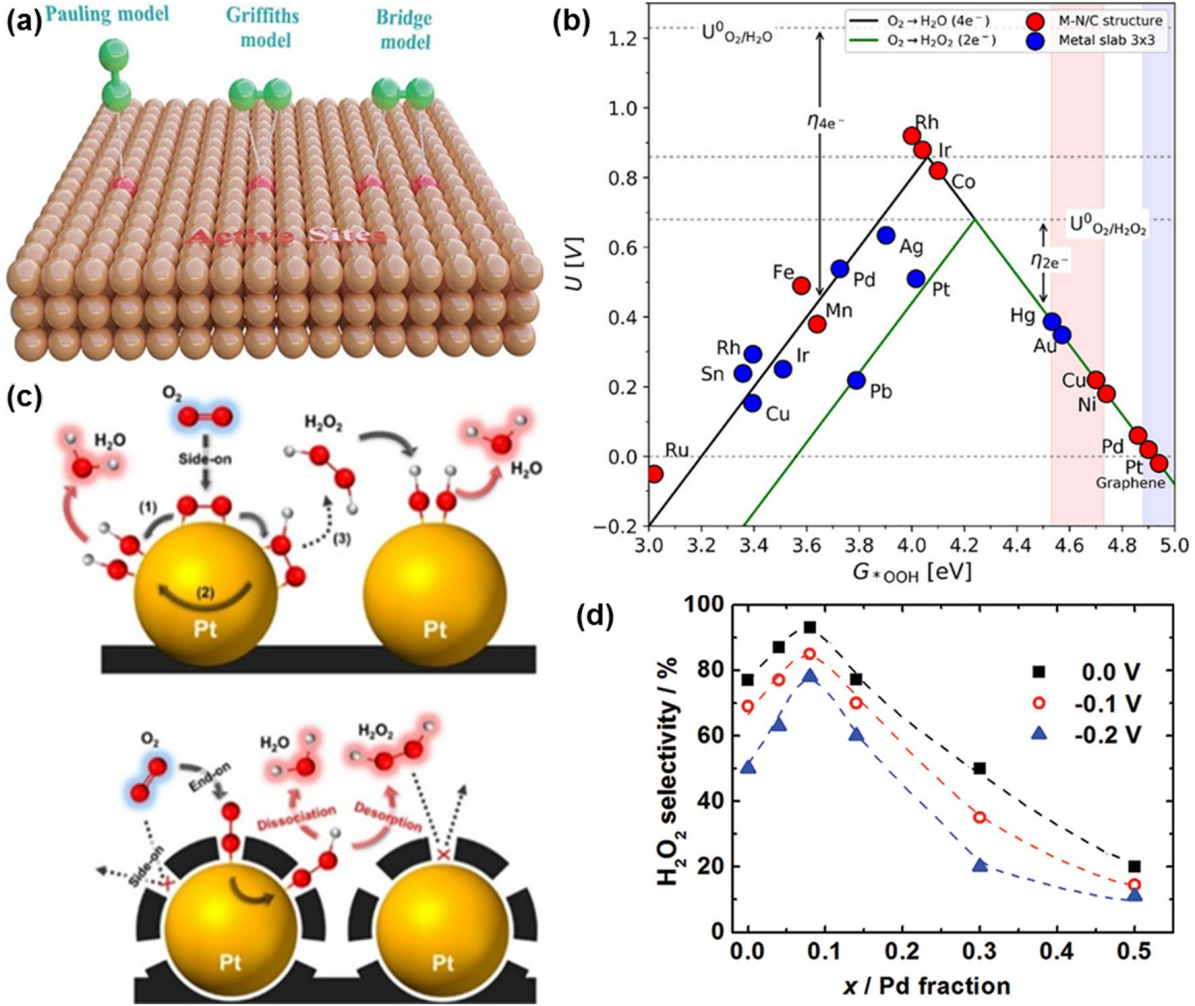
**a** Three typical modes for O_2_ adsorption. Reproduced with permission from Ref. [192]. Copyright 2021 Elsevier**. b** Sabatier volcano plots for electrochemical ORR for pure metal slabs and M–N/C. Reproduced with permission from Ref. [193]. Copyright 2018 American Chemical Society.** c** Proposed ORR pathways on the pristine Pt/C and carbon-coated Pt electrocatalysts. Reproduced with permission from Ref. [200]. Copyright 2014 American Chemical Society. **d** H_2_O_2_ selectivity of the Au_1‑x_Pd_x_ electrocatalyst with different palladium contents at potentials of 0, -0.1 and -0.2 V. Reproduced with permission from Ref. [208]. Copyright 2011 American Chemical Society

According to Sabatier’s principle, the binding energy between an OOH^*^ radical and an electrocatalyst should be neither too strong nor too weak for efficient H_2_O_2_ production, since an OOH^*^ radical is the only intermediate in the process of a 2e^−^ ORR pathway [41]. Meanwhile, the integrity of an O–O bond must be well maintained during the conversion process since the splitting of O–O bond is conductive to form water. It has to highlight here that the manners of oxygen adsorption on a catalyst mainly follow three modes (Fig. 12a), namely Pauling model (“end-on”), Griffiths model and Bridge model (“side-on”) [55, 192]. Only Pauling model is beneficial for the H_2_O_2_ generation via two-electron reduction of O_2_. This is due to its low influence on the O–O bond splitting. Differently, other two models induce the O–O bond splitting and facilitate the 4e^−^ ORR pathway. Note that it is more thermodynamically inclined to the pathway of the 4e^−^ ORR to form H_2_O in comparison with H_2_O_2_ generation via the 2e^−^ ORR pathway. To address this issue, the key is to design and synthesize advanced catalysts with high activity for O_2_ reduction, while well remaining O–O bond during the 2e^−^ ORR process. In the next section, the electrocatalysts on the selective reduction of O_2_ to H_2_O_2_ are reviewed in combination with some typical examples.

Single noble metal materials have been applied for electrocatalytic H_2_O_2_ synthesis. For example, DFT calculations have been firstly used to reveal the relationship between the ORR activity and various metallic catalysts. The Sabatier volcano plots were then established (Fig. 12b), namely the electrocatalytic ORR activity of different metals and metal-nitrogen/carbon (M–N/C) [193]. Taking the volcano curves of single metals (the left-hand side of the volcano diagram) as the example, the limiting potential of a 4e^−^ ORR process is more positive than that of a 2e^−^ ORR process, thermodynamically indicating that the 4e^−^ reduction reaction is more likely to occur to obtain H_2_O than the 2e^−^ reduction reaction to generate H_2_O_2_. While on the right-hand side of the volcano plots, the limiting potential of 2e^−^ and 4e^−^ ORR processes are located at the same position, which illustrates that they simultaneously take place to produce H_2_O_2_ and H_2_O, respectively. In more detail, as the cases that the strong interaction occurs between metals and O_2_ molecules, the selectivity of 2e^−^ ORR can be improved once the metal surface is modified, or its structure is regulated. Taking Pt metal as an example, O_2_ is adsorbed on the Pt surface mainly through a “side-on” mechanism, instead of the “end-on” mechanism. The H_2_O_2_ production on Pt catalysts is thus not conducive to be promoted [194]. In literature, the effects of the size and morphology of metal catalysts have been extensively investigated to explore the activity for electrocatalytic H_2_O_2_ production, namely via altering the O_2_ adsorption mode on the Pt electrocatalysts [195–197]. Note that the choice of suitable supports also modulates the selectivity of the Pt electrocatalysts toward electrocatalytic H_2_O_2_ production [198, 199]. For example, an amorphous carbon layer coated Pt catalyst was prepared by use of a chemical vapor deposition method with acetylene as the precursor [200]. This amorphous carbon layer presumably produces steric hindrance on the Pt surface, which induces O_2_ to be adsorbed through the end-on configuration, instead of side on configuration over the pristine Pt/C electrocatalyst (Fig. 12c). The 2e^−^ ORR pathway happened dominantly on the C(Pt)/C-3 h and C(Pt)/C-4 h electrocatalysts. Their H_2_O_2_ selectivity reached 41% at a potential of 0.1 V_RHE_. Similarly, sulfur-doped carbon using zeolite-templated was used to support catalyst, where this carbon material supported and dispersed 5 wt% Pt atoms [197]. This Pt atomic electrocatalyst followed a 2e^−^ ORR pathway. Its H_2_O_2_ selectivity was as high as 96%. Modified Pd species have been also designed and obtained to assess selective ORR [201, 202]. For instance, using a nano-Pd electrocatalyst, obtained through *in-situ* electrochemical deposition from Pd ions, high selectivity for the 2e^−^ ORR pathway to form H_2_O_2_ was achieved. Even when Pd is derived from the lowest precursor concentration, related nano-Pd electrocatalyst exhibited high selectivity, which was surpassed 95% [203].

In contrast, the noble metals (e.g., Au) that possess weak adsorption capability toward O_2_ molecules have been applied to reduce O_2_ for efficient, selective and stable H_2_O_2_ production [204–206]. Unfortunately, the adsorption of O_2_ molecules on the crystal plane of noble metals is different. Namely, the ORR pathway on different metal facets is different. For example, the too weak bond between an Au (111) surface and O_2_ molecules makes it difficult to form the OOH^*^ intermediate [207]. The H_2_O_2_ production on the Au (111) surface via the 2e^−^ ORR pathway exhibits a low efficiency and high energy consumption is associated. In this regard, the crystal plane of noble metals is applied as the reactive interface, since its binding energy with O_2_ molecule in a high index crystal plane is stronger than that occurred in an (111) facet. For example, an Au (211) facet effectively promotes the activity of a 2e^−^ ORR pathway for the H_2_O_2_ production.

Although the H_2_O_2_ selectivity of single metal electrocatalysts can be effectively adjusted via various means as discussed above, simultaneous improvement of the activity and selectivity for the 2e^−^ ORR pathway on single metal electrocatalysts is still challenging. This is mainly originated from the fact those with weaker interactions with O_2_ often need high overpotential to boost the 2e^−^ ORR, while those owning stronger interactions with O_2_ are prone to bring the direct the 4e^−^ ORR. Combining such metals (namely those weakly interact with O_2_ and those strongly interact with O_2_) to form metal alloys is thus a potential strategy to facilitate both activity and selectivity of metal electrocatalysts toward the H_2_O_2_ generation. For example, the Au_1-x_Pd_x_ nanoalloys supported on Vulcan XC-72 exhibited the superior selectivity toward the H_2_O_2_ production (nearly 95%) when the Pd content in the alloy arrived at 8%, surpassing that using single Au and Pd metal electrocatalysts (Fig. 12d) [208]. The Pd monomer surrounded by Au atoms on the Au–Pd nanoalloy surface was suggested to promote the H_2_O_2_ formation via an “end-on” O_2_ adsorption model. When the percentage of Pd was increased to 15%, the selectivity of the H_2_O_2_ generation was decreased. This is because the presence of continuous Pd atoms (e.g., two adjacent Pd atoms) altered the O_2_ adsorption model and facilitated the H_2_O formation. In a theoretical work, the tactics was provided based on DFT calculations to screen for metal alloy electrocatalysts that feature excellent activity toward electrocatalytic H_2_O_2_ production [5]. These metal alloys are prepared by surrounding individual elements (e.g., Pt, Ir, Pd, Rh and Ru) that are active for the ORR by inert metallic elements (e.g., Hg and Au). Since the active elements exhibit strong adsorption toward O_2_ molecule, these metal alloys can effectively reduce O_2_ to OOH^*^. On the other side, they do not break the O–O bond, because of their difficulty in dissociating OOH^*^ in combination with the neighboring inert sites. In short, the activity and selectivity of metal alloy catalysts toward the 2e^−^ ORR can be regulated by using the advantages of the isolated active sites. Theoretical models have even predicted that the isolated active site of the Pt-Hg alloy can effectively activate O_2_ molecules and boost the H_2_O_2_ synthesis. Experimentally, several Hg-based bimetallic alloys have shown high activity and selectivity for the H_2_O_2_ production [209]. Among them, the Pd-Hg alloy exhibited the highest current for the H_2_O_2_ production at the same overpotential, as predicted from the DFT calculations. In more detail, the activities of Pt-Hg, Ag and Ag-Hg alloys are one order of magnitude higher than that of individual Au electrocatalyst, while the activity of a Pd-Hg alloy is two orders of magnitude higher than that of individual Au electrocatalyst. The AuPd alloy nanoparticles have been used to disclose the relationship between the activity of electrocatalytic H_2_O_2_ production and molar ratio of Au to Pd. Modulating the composition of alloy catalysts is thus recognized as a new way to promote the 2e^−^ ORR for electrocatalytic H_2_O_2_ production [210].

#### Carbon-based Electrocatalysts

**Fig. 13:**
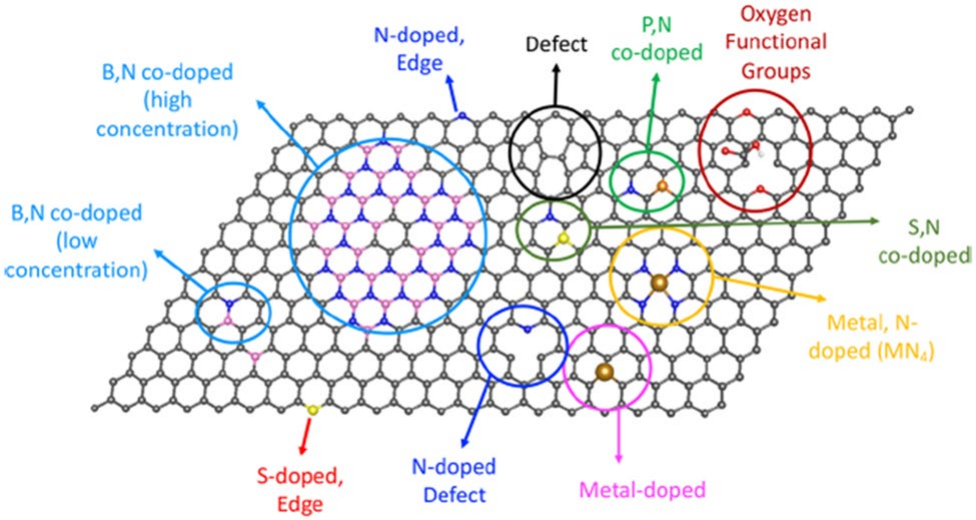
Schematic demonstration of tuning electronic structures of carbon electrocatalysts for electrocatalytic H_2_O_2_ production via the 2e^−^ ORR pathway. Reproduced with permission from Ref. [18]. Copyright 2020 American Chemical Society

It is clear that the noble metals and their alloys feature high activity and selectivity for electrocatalytic H_2_O_2_ synthesis via the 2e^−^ ORR pathway. Unfortunately, they are expensive and scarce, hindering their large-scale applications. Many advanced electrocatalysts with reduced costs have been thus explored for electrocatalytic H_2_O_2_ synthesis. Carbon-based electrocatalysts have been considered to be one of the most promising substitutes of noble metal-based catalysts. This is due to their unique surface and structural properties, abundant availability and low cost for their synthesis [211–214]. Note that the pristine carbon materials only exhibit low catalytic activity for the 2e^−^ ORR, thus the reconstruction of carbon materials or their surface modification (Fig. 13) have been developed to optimize their electronic properties for electrocatalytic H_2_O_2_ generation [18]. The widely reported approaches include manufacturing porous structures and defects, heteroatom doping and surface (oxygen) functionalization are provided.

**Fig. 14:**
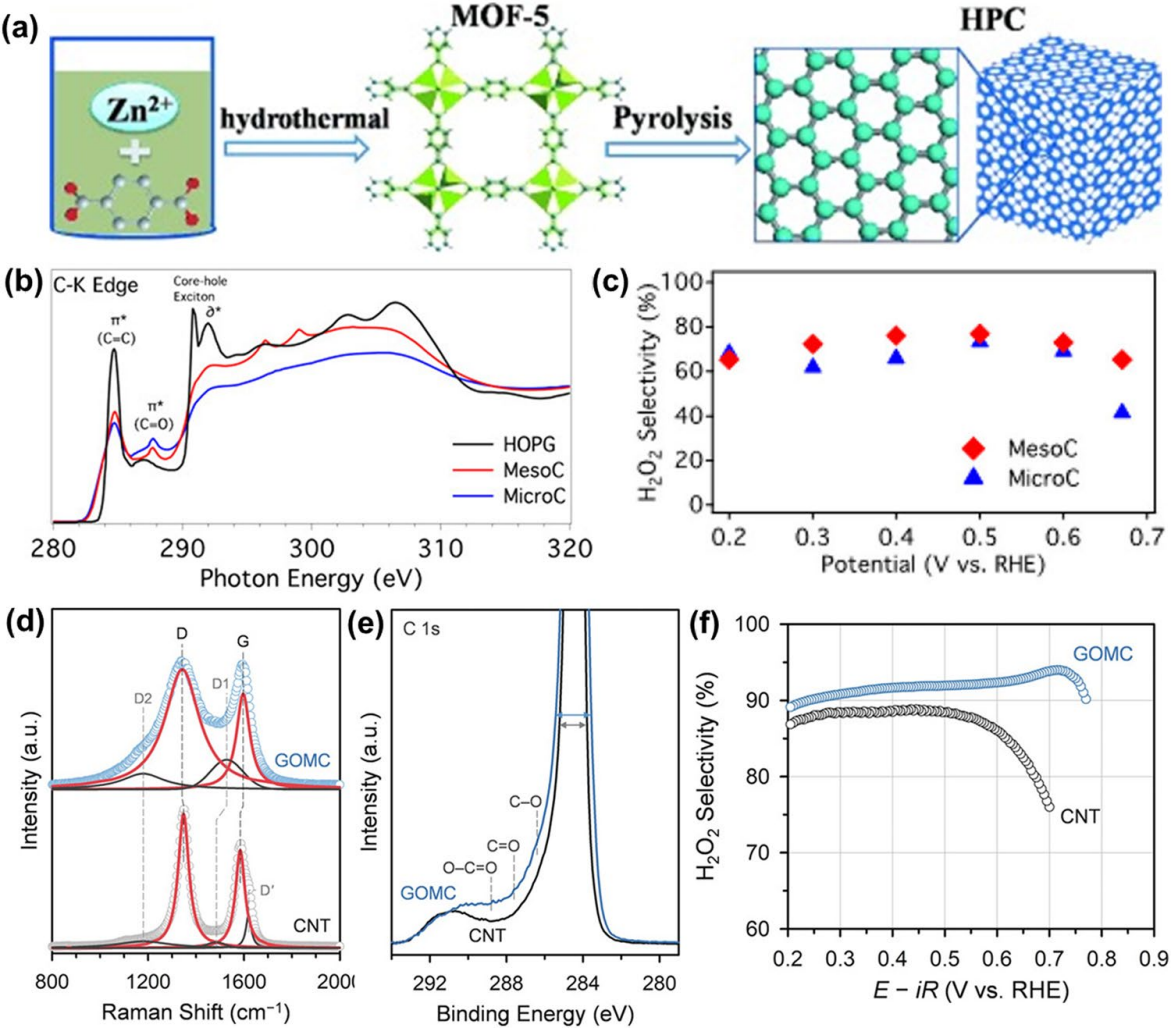
**a** Schematic illustration of the synthetic route to HPC. Reproduced with permission from Ref. [215]. Copyright 2015 Wiley–VCH Verlag GmbH & Co. KGaA, Weinheim. **b** NEXAFS spectra of the C-K edge in HOPG, MesoC and MicroC. **c** H_2_O_2_ selectivity of the MesoC and MicroC electrocatalysts. Reproduced with permission from Ref. [217]. Copyright 2018 American Chemical Society. **d** Raman spectra, **e** C 1*s* XPS spectra and **f** H_2_O_2_ selectivity of GOMC and CNT. Reproduced with permission from Ref. [218]. Copyright 2019 Wiley–VCH Verlag GmbH & Co. KGaA, Weinheim

Among diverse carbon materials, porous and defective carbon materials are highly suitable for the 2e^−^ ORR. This is due to their unique advantages, such as their high surface areas, tunable porosities and high electronic conductivities. It has been widely recognized that carbon electrocatalysts with a porous structure can provide abundant active sites and shorten diffusion distance for the reactive species, producing positive contributions for their electrocatalytic H_2_O_2_ generation. For example, various hierarchically porous carbon (HPC), produced by hydrothermal treatment and subsequent carbonization, have been applied for selective H_2_O_2_ formation (Fig. 14a) [215]. The HPC-H24 (hydrothermal treatment for 24 h and carbonization under H_2_) exhibited the most positive peak potential and the highest selectivity for the 2e^−^ ORR, namely the best performance of selective H_2_O_2_ formation. The remarkable electrocatalytic performance of HPC-H24 was assumed to be originated from high contents of active sites (e.g., *sp*^3^-C bonds, vacancy defects and edge defects), more exposed and accessible active sites and fast mass transport of H_2_O_2_ inside a porous structure. On the contrary, it was acknowledged that edge-rich graphene presented the typical 4e^−^ ORR mechanism [216]. Later, two different types of porous carbon, namely microporous carbon (denoted as MicroC) and mesoporous carbon (denoted as MesoC) have been developed toward the H_2_O_2_ production in alkaline conditions (Fig. 14b) [217]. From the broad π^*^ resonance of near-edge X-ray absorption fine structure (NEXAFS) spectra, the conclusion that both MicroC and MesoC have a larger number of *sp*^2^ carbon defective sites than HOPG has been drawn. Electrochemical experiments suggested that both MicroC and MesoC had superior activity and selectivity for the 2e^−^ ORR, where the onset potential was very close to the thermodynamic equilibrium potential (0.7 V_RHE_), and the selectivity of the H_2_O_2_ production even outperformed 70% in some cases (Fig. 14c). In addition, DFT calculations have illustrated the *sp*^2^-type defects are the active sites for O_2_ reduction, which is beneficial for the H_2_O_2_ generation. Similar optimization of defects and pore size in carbon materials have been reported for electrocatalytic H_2_O_2_ production. For example, the graphitic carbon with rich edge sites has been applied as an electrocatalyst to promote the H_2_O_2_ synthesis. Its activity was about 28 times higher than that of a basal plane-rich carbon nanotube (CNT) [218]. This enhanced activity was explained by the fact that graphitic ordered mesoporous carbon (GOMC) has a larger portion of defective carbon and reaction sites (*sp*^2^-C) when compared with CNT. Such a statement was solidly supported from the experimental results by means of Raman spectroscopy (Fig. 14d) and XPS (Fig. 14e) characterization. Compared with CNTs, the GOMC showed the H_2_O_2_ selectivity higher than 90% via the 2e^−^ ORR pathway and excellent stability (e.g., 90% of the initial current is retained) (Fig. 14f).

**Fig. 15:**
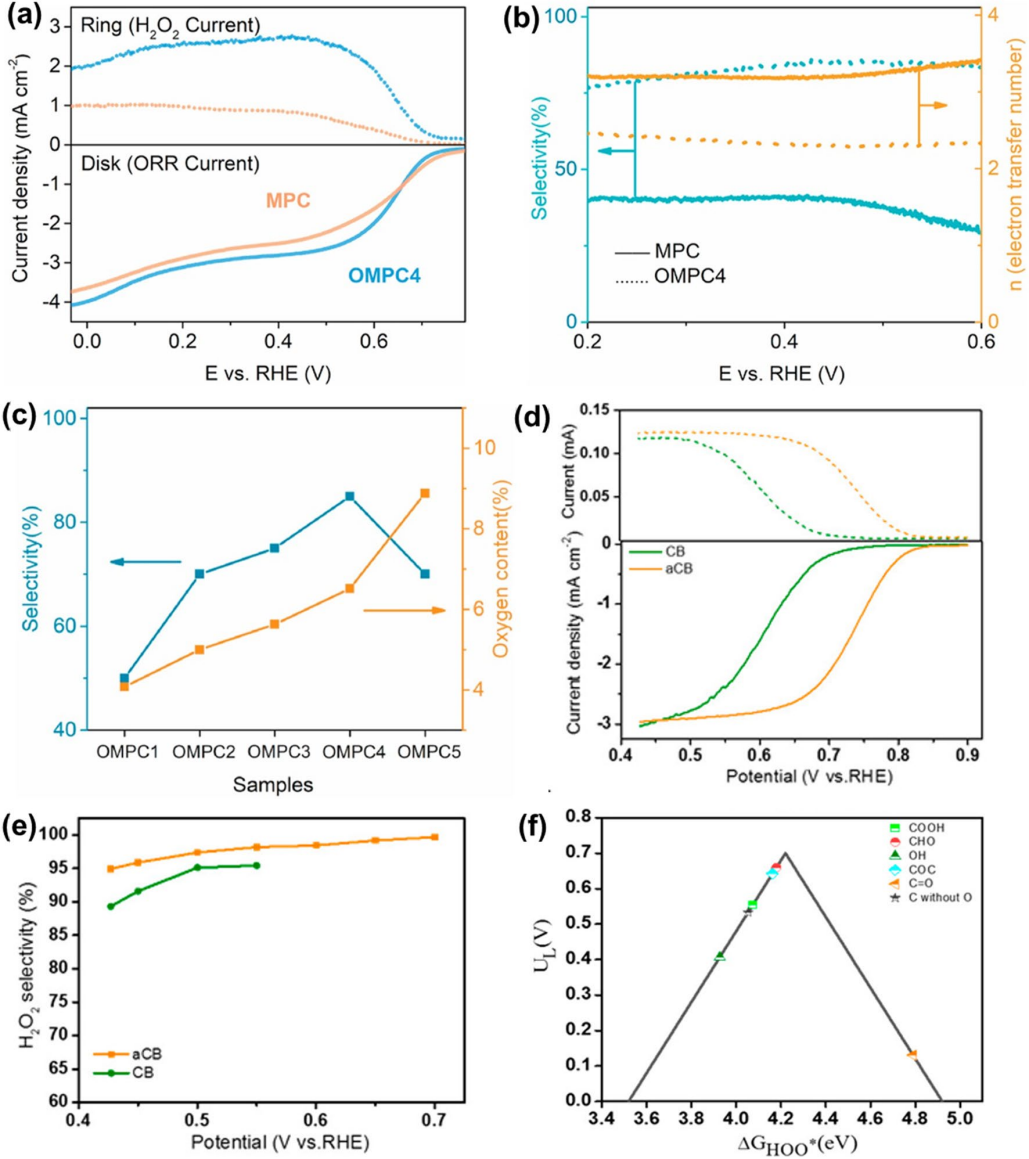
**a** ORR performance, **b** selectivity and electron transfer number of OMPC4 and MPC in O_2_-saturated 0.1 M KOH. **c** Relationship between the selectivity and oxygen content of various OMPC-samples. Reproduced with permission from Ref. [220]. Copyright 2020 Elsevier. **d** ORR performance and **e** selectivity of aCB and CB in 0.1 M O_2_-saturated KOH. **f** Activity volcano plot of the 2e^−^ ORR. Reproduced with permission from Ref. [222]. Copyright 2018 American Chemical Society

It has been reported that oxygen-containing groups play an important role on the activity and selectivity of carbon catalysts toward the 2e^−^ ORR [219–225]. For instance, oxygen-doped microporous polypyrene carbons (OMPC) exhibited the enhanced activity and selectivity for electrocatalytic H_2_O_2_ generation [220]. In basic electrolyte, the OMPC4 (oxygen content is 6.52 at%) showed obviously stronger ability to H_2_O_2_ production from the *I*_ring_ than MPC, as well as the higher limited oxygen reduction current (*I*_disk_) (Fig. 15a). Meanwhile, the selectivity of the H_2_O_2_ production on OMPC-4 was remarkably increased (e.g., from 40 to 87% at 0.42 V_RHE_) and the calculated electron transfer number (*n*) was close to 2 through oxygen doping on OMPC (Fig. 15b). In addition, the selectivity of MPC with the different oxygen content is displayed in Fig. 15c, where OMPC-4 exhibited the highest value. In another case, oxygenated carbon (namely activated carbon black, denoted as aCB) electrocatalyst showed higher ORR activity meaning the stronger capacity of H_2_O_2_ generation than CB (Fig. 15d) [222]. The selectivity above 94% was obtained on the aCB catalyst in the potential range from 0.4 to 0.7 V as depicted in Fig. 15e. The DFT calculations further disclosed the activity difference of these oxygen-containing groups toward the H_2_O_2_ production (Fig. 15f) is originated from the fact that C–O–C and -CHO are the most active sites toward the H_2_O_2_ synthesis compared to other functional groups.

**Fig. 16:**
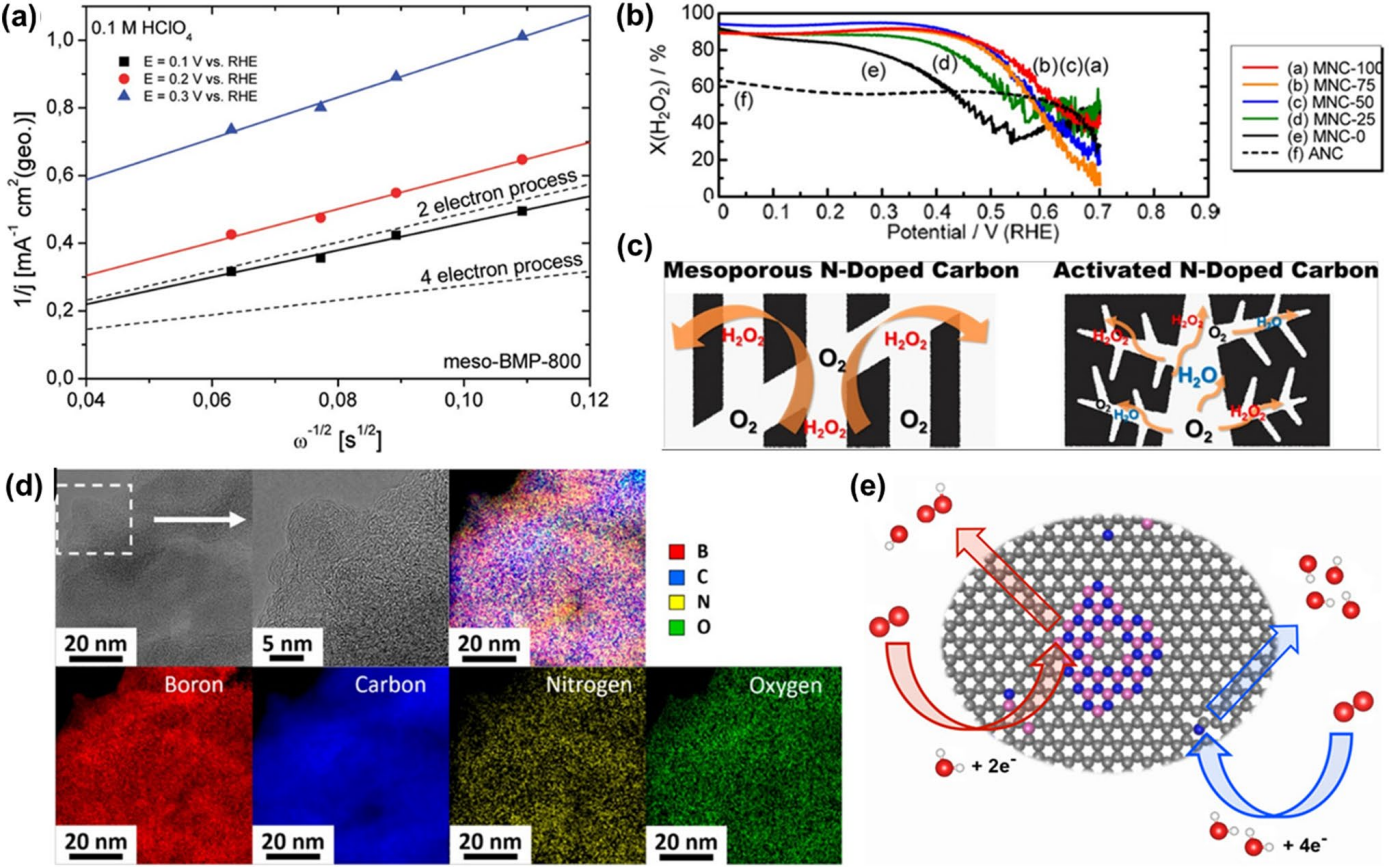
**a** The number of electrons transferred based on the Koutecky–Levich plot. Reproduced with permission from Ref. [236]. Copyright 2012 American Chemical Society.** b** RRDE voltammograms toward ORR over the MNCs and ANC. **c** Schematic illustration of the ORR on different N-doped carbon. Reproduced with permission from Ref. [237]. Copyright 2014 American Chemical Society.** d** HRTEM and elemental mapping images of BN-C1. **e** Schematic illustration of the types of doping motifs and ORR mechanisms on these active sites in the B,N co-doped electrocatalysts (C: gray, N: blue, B: red; O: white). Reproduced with permission from Ref. [241]. Copyright 2018 American Chemical Society

In addition to structured carbon materials, heteroatom (e.g., N, S, B) doped carbon materials have been widely studied for the electrocatalytic H_2_O_2_ production via the 2e^−^ ORR pathway [226–230]. Many studies have confirmed that N-doping of a carbon material can effectively promote the catalytic performance for the ORR, because the higher electronegativity of N atoms than carbon atoms can activate π electrons in carbon system. The charge redistribution is then induced or the electronic properties of carbon materials are changed, leading to varied adsorption capacity of carbon catalysts toward O_2_ and generated intermediates [55, 231, 232]. It must be pointed out that common N-doped carbon catalysts are conducive to the 4e^−^ ORR pathway to produce water [233–235]. It is thus extremely important to bridge the relationship of a nano-structured carbon catalyst and the content of doped N inside with the ORR selectivity. For example, a simple hard-templating strategy was adopted to obtain mesoporous nitrogen-doped carbon (meso-BMP-800) catalyst and its nitrogen content was 14.2 wt% (as determined from XPS analysis) or 17.17 wt% (as evaluated from elemental analysis) [236]. On this electrocatalyst, selective H_2_O_2_ production was enhanced (Fig. 16a**).** This 2e^−^ ORR process has been experimentally and theoretically doable. In such situations, the strategy of N-doping of carbon materials helped to achieve high conductivities of carbon materials and their high selectivity for electrocatalytic H_2_O_2_ production. It is so-called a pure two-electron mechanism. However, the regulatory mechanism of the structure of a carbon catalyst on its reactivity is still unclear. In this context, the effect of porous structures of carbon catalysts on their catalytic properties toward the H_2_O_2_ production has been explored [237]. Mesoporous nitrogen-doped carbon (MNC) exhibited higher H_2_O_2_ selectivity (> 90%, Fig. 16b) than activated nitrogen-doped carbon (ANC). Enhanced mass transport into a mesoporous structure of this catalyst was suggested to be responsible for such high H_2_O_2_ selectivity of MNC (Fig. 16c). Later, systematic studies have been explored to reveal the correlations between the performance of electrocatalytic H_2_O_2_ production on carbon catalysts and their structural characters and surface physicochemical properties [238]. The selectivity of the H_2_O_2_ production on N-doped carbon catalysts is more dependent on the nitrogen doping effect rather than zeta potentials and defect sites of used carbon catalysts. In other words, the tuning of N-doping in carbon materials is very important and even more critical than structuring of carbon materials to obtain the high performance for electrocatalytic H_2_O_2_ production. In addition to N-doped carbon materials, B/S-doped ones were synthesized for electrocatalytic H_2_O_2_ generation. For example, B-doped mesoporous carbon materials have been synthesized via a convenient hydrothermal method, where F127 and boric acid were the template and boron source, respectively [239]. The obtained B-MC-F2 exhibited enhanced performance for electrocatalytic H_2_O_2_ formation in alkaline solution. In another case, a hollow, porous and S-doped carbon spheres presented higher activity and selectivity (> 70%) via the 2e^−^ ORR pathway than carbon spheres without S-doping. The introduced S–S bond is believed to feature low overpotential for the 2e^−^ ORR, leading to high activity for electrocatalytic H_2_O_2_ production [240]. In addition to the monoatomic-doped carbon materials, multiple heteroatoms co-doped carbon materials have been utilized for electrocatalytic H_2_O_2_ generation. For example, B,N co-doped (BN-C) carbon materials prepared through a simple heat-treatment (Fig. 16d**)** showed higher activity and selectivity for the H_2_O_2_ production via the 2e^−^ ORR pathway than the N-doped carbon materials [241]. Once again, there are various facts (e.g., heteroatom doping) affect the 2e^−^ and 4e^−^ pathways on carbon materials (Fig. 16e). However, the H_2_O_2_ formation on B,N-doped carbon materials was preferentially driven by 2e^−^ ORR pathway, resulting from unique catalytic behavior at the interface of h-BN domains and the host graphene lattice.

**Fig. 17:**
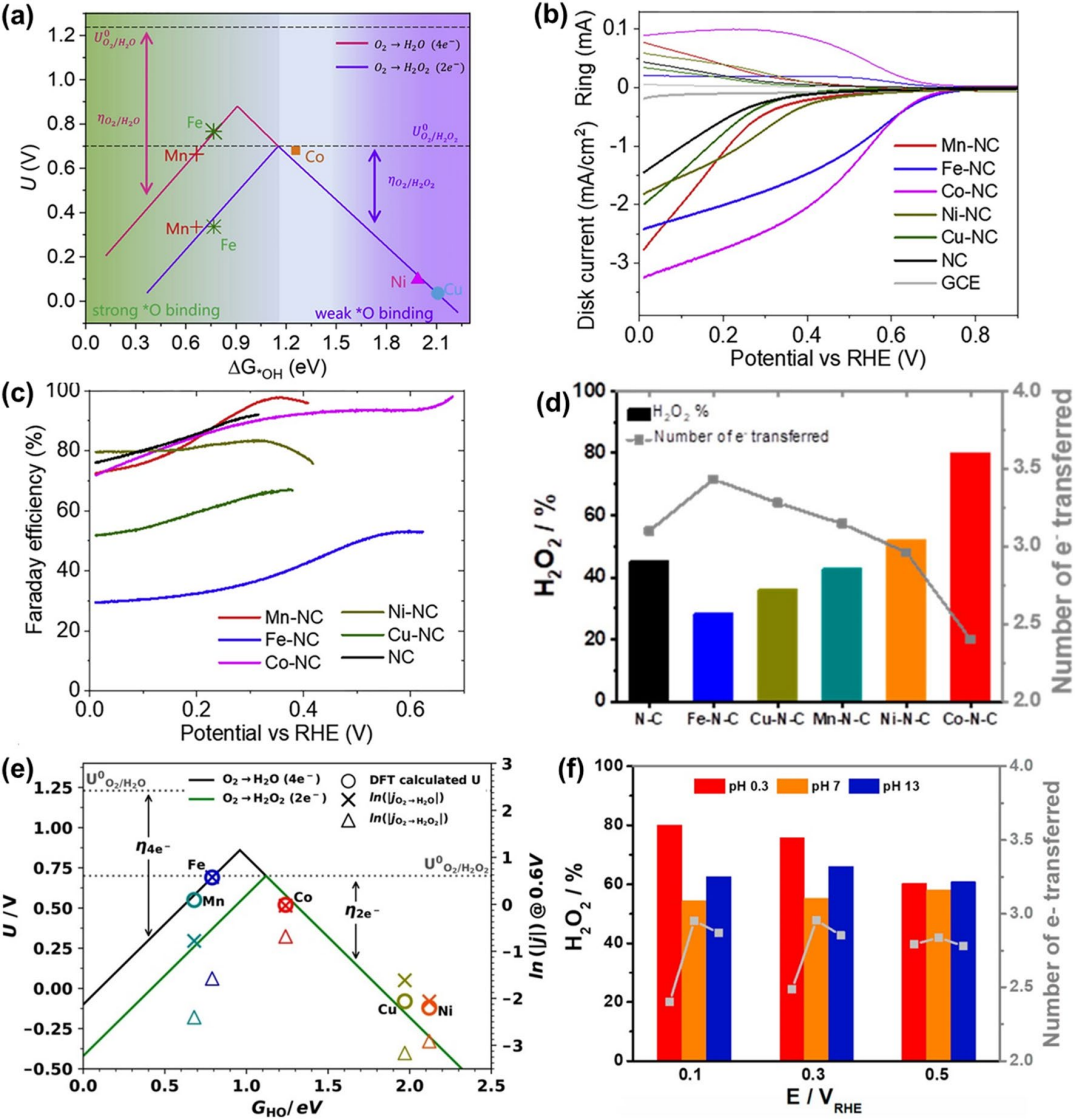
**a** Activity-volcano plot of ORR through 2e^−^ or 4e^−^ route. **b** Linear sweep voltammograms (LSVs) of various catalysts in O_2_-saturated 0.1 M HClO_4_. **c** Faradic efficiencies of the H_2_O_2_ formation at different potentials. Reproduced with permission from Ref. [246]. Copyright 2019 Elsevier. **d** H_2_O_2_ selectivity and the number of electrons transferred (*n*) at +0.1 V_RHE_. **e** Activity-volcano plot for the ORR. **f** H_2_O_2_ selectivity and the number of electrons transferred (*n*) at different potentials. Reproduced with permission from Ref. [247]. Copyright 2019 American Chemical Society

As an interesting type of electrocatalysts, N-doped carbon materials with atomically dispersed transition metals (M–N-C) have been paid close attention in recent years for various catalytic applications, especially as unique catalytic systems for the selective H_2_O_2_ production via the 2e^−^ ORR pathway [242–245]. For example, the relation between physicochemical properties of transition metal (e.g., Mn, Fe, Co, Ni and Cu) single-atom anchored in N-doped graphene and their performance of electrocatalytic H_2_O_2_ production has been systematically explored by the means of theoretical calculations and experimental tools [246]. The DFT calculations indicated that the intermediates of ^*^OOH, ^*^O and ^*^OH that are adsorbed on the top site of the metal (M) atoms are the most stable configurations. According to related activity-volcano plot (Fig. 17a), Ni and Cu single-atom catalysts (SACs) tend to follow the 2e^−^ ORR pathway during O_2_ reduction, suggesting their high selectivity toward the H_2_O_2_ generation. On the other side, a large barrier exists between the reduction of ^*^OOH and the activation of O_2_ molecule. A large overpotential for the ORR is thus indispensable, suggesting low activity of these SACs for electrocatalytic H_2_O_2_ production. Differently, the 4e^−^ ORR pathway becomes more favorable on the Mn and Fe SACs during the ORR, resulting in poor selectivity for electrocatalytic H_2_O_2_ production. Remarkably, only Co SACs exhibit optimal d-band centers near the apex of the volcano plots, indicated the appropriate adsorption for the intermediates to facilitate the 2e^−^ ORR pathway. Its high activity and selectivity for the 2e^−^ ORR were confirmed by electrochemical ORR tests, where this Co-NC catalyst showed high activity and selectivity for the H_2_O_2_ production in acidic media (Fig. 17b, c). Similarly, the activity and selectivity of M–N–C (M = Mn, Fe, Co, Ni and Cu) SACs for electrocatalytic H_2_O_2_ production have been investigated [247]. Among these catalysts, the Co–N–C catalyst exhibited the highest ORR selectivity (80%) at 0.1 V_RHE_ in 0.5 M H_2_SO_4_ than that of Fe–N–C (28%), Cu–N–C (36%), Mn–N–C (43%), N–C (45%) and Ni–N–C (52%) catalysts under the same conditions. Such high performance was assigned to the fact that the binding free energy of Co–N–C is close to that at the top of the H_2_O_2_ production in the volcano plot (Fig. 17e). The number of electrons transferred of a Co–N–C catalyst was much closer to 2 (Fig. 17d). This result indicates that the Co–N–C catalyst is more favorable for 2e^−^ ORR pathway to produce H_2_O_2_ (Fig. 17f). The H_2_O_2_ selectivity of a Co–N–C catalyst is increased with the potential decreased in acidic solutions, while remained well in the whole potential range under neutral and alkaline electrolyte. One optimal Co–N_4_ moiety incorporated in N-doped graphene exhibited excellent H_2_O_2_ productivity and durability [248]. In the volcano plot of ORR activity, none of M-N_4_/graphene catalysts are located at the peak position (Δ*G*_OOH*_ = 4.2 eV) for the H_2_O_2_ production. As for the Co-N_4_, its Δ*G*_OOH*_ can be modulated once functional groups are attached. For example, when one O* is adsorbed near the Co-N_4_ moiety (denoted as Co–N_4_(O)), the Δ*G*_OOH*_ value increases from 3.9 to 4.1 eV, very close to the optimal Δ*G* value for the H_2_O_2_ production. This phenomenon can be clarified according to the difference in the charge states of cobalt atoms. Specifically, the charge states of cobalt atoms in the Co–N_4_(O) and Co–N_4_(2O) moieties are 0.05 e^−^ and 0.10 e^−^ more positive than Co–N_4_. Therefore, a reasonable design strategy of electrocatalytic H_2_O_2_ synthesis is to slightly enlarge Δ*G*_OOH*_ value of the Co–N_4_/graphene electrocatalyst by constructing electron-rich oxygen species near the Co–N_4_ moiety. In this way, the charge states of cobalt atoms are thus gently increased. With aid of this strategy, the Co_1_-NG(O) SAC were prepared. Among them, the Co_1_-NG(O) catalyst showed excellent activity and high selectivity for electrocatalytic H_2_O_2_ generation.

### Electrocatalytic H_2_O_2_ Synthesis Through a 2e^−^ WOR Pathway

In addition to the 2e^−^ ORR pathway, the 2e^−^ WOR has been considered as another promising approach toward electrocatalytic H_2_O_2_ production. It is worth mentioning that in a 2e^−^ WOR process only H_2_O is involved as the reactant for the electrocatalytic H_2_O_2_ production. Such systems are thus more convenient to be operated than those using the 2e^−^ ORR pathway. However, the H_2_O_2_ generation via the 2e^−^ WOR pathway usually displays relatively low Faradaic efficiency. This is because the competitive 4e^−^ WOR (namely O_2_ evolution process) is thermodynamically more favorable than the 2e^−^ WOR (namely the H_2_O_2_ formation) accompanying with the autocatalytic decomposition from H_2_O_2_ into O_2_. In this regard, there are limited studies on this infant field.

#### Metal Oxides

**Fig. 18:**
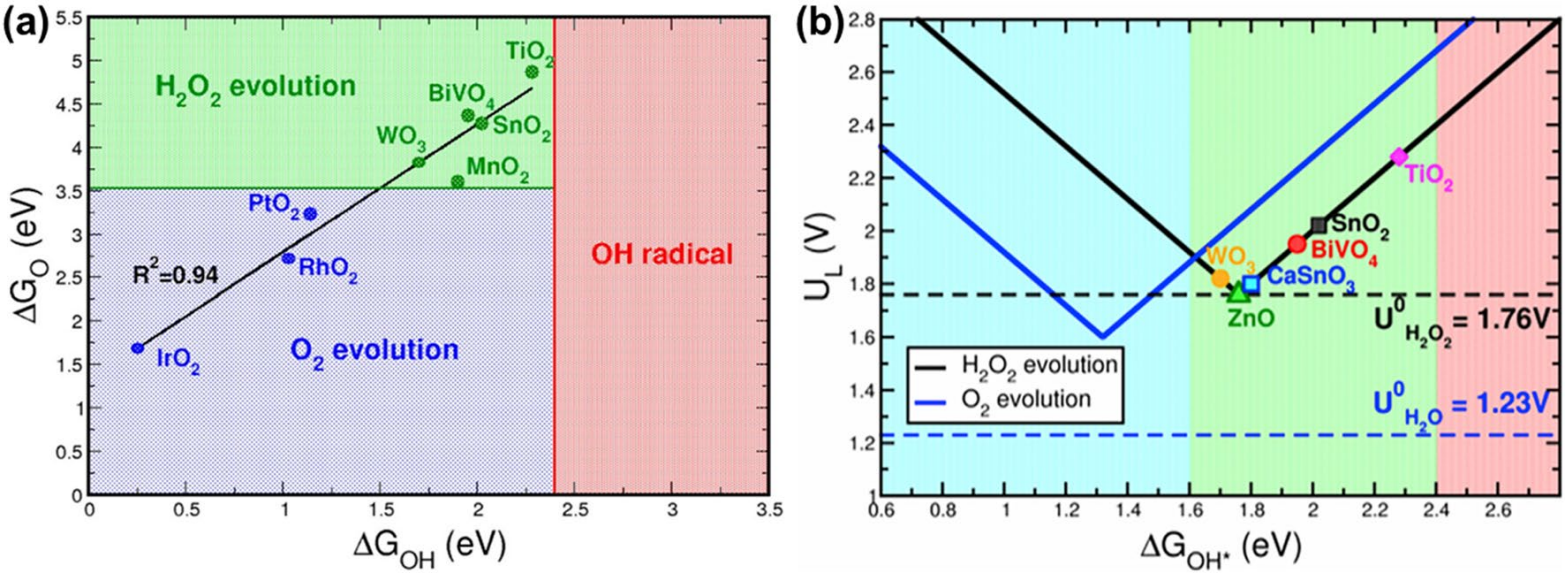
**a** Phase diagram represented by the binding energies of O* and OH*. Reproduced with permission from Ref. [261]. Copyright 2017 American Chemical Society.** b** Activity volcano plots for the 2e^−^ and 4e^−^ WOR. Reproduced with permission from Ref. [54]. Copyright 2020 Elsevier

Noble metal oxides (e.g., RuO_2_ and IrO_2_) have been frequently applied for various catalytic applications, due to their high catalytic activities and long-term stability at high potentials [257, 258]. However, their prices are high to limit the large-scale applications [259]. Many alternative noble-free metal oxides thus became more popular in the H_2_O_2_ production via the 2e^−^ WOR pathway (Table 4). For example, the free energies of OH* and O* were identified as key parameters to confirm selectivity and activity of different electrocatalysts toward the 2e^−^ WOR [260]. The metal oxide catalysts that simultaneously meet two conditions for OH* and O* binding energies (Δ*G*_O_ ≳ 3.5 eV and Δ*G*_OH_ ≲ 2.4 eV) are favorable for selective H_2_O_2_ production [261]_._ In the summarized relationship between the selectivity of 2e^−^ WOR and the binding energies of O/OH for a number of different metal oxides (Fig. 18a**)**, metal oxides (e.g., WO_3_, BiVO_4_, MnO_2_ and SnO_2_) are located in the region for weak O adsorption energies (green region). They thus promote the 2e^−^ WOR to form H_2_O_2_. In verse, metal oxides (e.g., IrO_2_, RhO_2_ and PtO_2_) that feature strong O adsorption energies (blue region) are favorable to generate O_2_ as the major product. This study provides a good theoretical guide to rationally design metal oxide catalysts in the process of 2e^−^ WOR to promote the H_2_O_2_ production. Meanwhile, the activity trends of various metal oxides (e.g., WO_3_, ZnO, CaSnO_3_, BiVO_4_, SnO_2_ and TiO_2_) for the H_2_O_2_ production via the WOR pathway were summarized as the activity volcano plots. They were based on the relationship between limiting potentials and the free energy of OH* (Fig. 18b) [54]. It was proved again that the catalysts possessing suitable Δ*G*_OH*_ value (e.g., from 1.6 to 2.4 eV) and low overpotential for the 2e^−^ WOR exhibits high selectivity and activity toward the H_2_O_2_ production.

**Table 4:** Summary of electrocatalytic H_2_O_2_ production via a 2e^−^ WOR pathway

Catalyst	Electrolyte	Faraday efficiency (%)	Onset potential (V_RHE_)	Refs
MnO_x_	1 M BAS	77	1.91 (1.0 mA cm^−2^)	[50]
ZnO	2 M KHCO_3_	81 at peak potential	1.80 (0.1 mA cm^−2^)	[268]
C, N codoped TiO_2_	0.05 M Na_2_SO_4_	8 (2.9 V_Ag/AgCl_)	2.66 V_Ag/AgCl_ (0.1 mA cm^−2^)	[270]
BiVO_4_/FTO	2.0 M KHCO_3_	35	∼2.50 (0.1 mA cm^−2^)	[271]
CaSnO_3_	2 M KHCO_3_	76 (3.2 V_RHE_)	1.99 (0.2 mA cm^−2^)	[267]
SnO_2_	2 M KHCO_3_	32 (3.2 V_RHE_)	2.13 (0.2 mA cm^−2^)	
4:1 (Ti,Mn)O_x_	0.5 M pH = 7 phosphate buffer	98 (0.1 mA cm^−2^)	1.89 (0.2 mA cm^−2^)	[269]
Gd-Doped BiVO_4_ (6% Gd:BVO)	2 M KHCO_3_	78 (~ 3.2 V_RHE_)	2.15	[264]
Bi_2_WO_6_:5%Mo	2 M KHCO_3_	79 (3.2 V_RHE_)	–	[265]
Nanoneedles BiVO_4_	1 M KHCO_3_	–	2.25 (2.5 mA cm^−2^)	[263]
Al_2_O_3_(CVD5)/BiVO_4_/FTO	0.5 M KHCO_3_	97 (*I* = 2 mA)	–	[272]
BiVO_4_	1 M NaHCO_3_	70 (3.1 V_RHE_)	∼1.90 (0.2 mA cm^−2^)	[262]

**Fig. 19:**
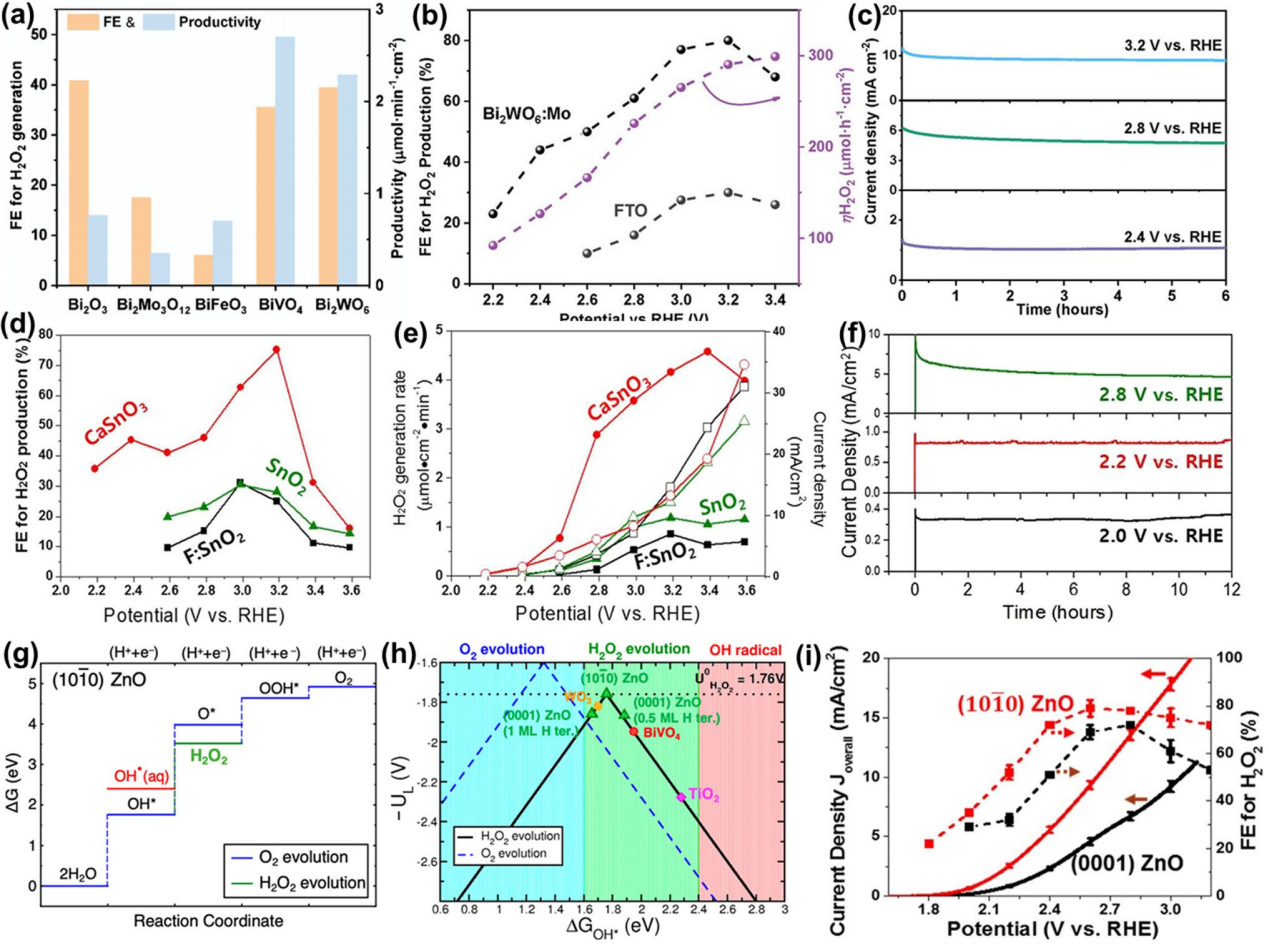
**a** Faradaic efficiencies (FEs) and productivity of Bi-based oxides at 2.6 V_RHE_. **b** FE and H_2_O_2_ generation rate of a FTO/Bi_2_WO_6_:5%Mo electrocatalyst at various potentials. **c** Stability measurement of a FTO/Bi_2_WO_6_:5%Mo electrocatalyst under different potentials. Reproduced with permission from Ref. [265]. Copyright 2020 Wiley–VCH GmbH. **d** FE, H_2_O_2_ generation rates and **e** the densities of different catalysts at different potentials. **f** Durability test of CaSnO_3_ catalyst under various potentials. Reproduced with permission from Ref. [267]. Copyright 2019 American Chemical Society. **g** Free energy diagram of the WOR for the most active (10 $\overline{1}$ 0) ZnO catalyst. **h** Volcano plots of 4e^−^ WOR to O_2_ and 2e^−^ WOR to H_2_O_2_ as a function of OH* binding free energies (Δ*G*_OH*_). **i** Overall current density and FE for H_2_O_2_. Reproduced with permission from Ref. [268]. Copyright 2019 American Chemical Society

In 2012, a MnO_x_ electrocatalyst was applied for the H_2_O_2_ formation via the 2e^−^ WOR pathway [50]_._ In this system, 77% of the H_2_O_2_ production was achieved. Unfortunately, the activity and yield of H_2_O_2_ were too low. This low efficiency was attributed to the competing process of the 4e^−^ ORR and/or continuous loss of H_2_O_2_ from the disproportionation. Three BiVO_4_ catalysts with different morphologies containing different surface ratios of (−121) and (040) facets (so-called seed, nanoneedles and truncated BiVO_4_) have been applied for electrocatalytic H_2_O_2_ production [263]. The high-index plane (−121) was disclosed to be favorable for the H_2_O_2_ formation in 1 M KHCO_3_ electrolyte rather than the O_2_ formation. Unfortunately, BiVO_4_ has poor stability in H_2_O_2_ synthesis [264]. The Bi_2_WO_6_ catalysts were then selected as alternatives to BiVO_4_ for the H_2_O_2_ generation via the 2e^−^ WOR pathway [265]. Among a series of Bi-based oxides (Fig. 19a), Bi_2_O_3_, BiVO_4_ and Bi_2_WO_6_ displayed preference for electrocatalytic H_2_O_2_ production. On the Bi_2_WO_6_ electrocatalyst, the best performance for H_2_O_2_ generation was achieved. The current density on the Bi_2_WO_6_ electrocatalyst was further increased by means of optimizing the morphology and Mo doping of the Bi_2_WO_6_ electrocatalyst. The faradaic efficiency of a FTO/Bi_2_WO_6_:5%Mo electrocatalyst was 79% at 3.2 V_RHE_ and its maximum yield rate of electrocatalytic H_2_O_2_ generation rate was as high as 300 μmol h^−1^ cm^−2^ at 3.4 V_RHE_ (Fig. 19b**)**. The durability test of this electrocatalyst is very stable for such electrocatalytic H_2_O_2_ production (Fig. 19c).

Other metal oxides such as CaSnO_3_ and ZnO have been also used for the H_2_O_2_ production via the 2e^−^ WOR pathway [266–268]. For example, CaSnO_3_ nanoparticles, prepared via the colloidal synthesis method, have been employed to evaluate H_2_O_2_ production in 2 M KHCO_3_ electrolyte [267]. Its Faraday efficiency (FE) was 76% at 3.2 V_RHE_, higher than that of F:SnO_2_ and SnO_2_ (Fig. 19d**)**. The H_2_O_2_ generation rates are outstanding at applied potentials (Fig. 19e**)**. Meanwhile, it exhibited excellent stability during this 2e^−^ WOR process at 2.0 V_RHE_ within 12 h. Slight decrease occurred when a potential of at 2.2 or 2.8 V_RHE_ is applied (Fig. 19f). In another case, metal oxide of ZnO was identified as an ideal catalyst for electrocatalytic H_2_O_2_ synthesis. The (10 $\overline{1}$ 0) facet on the ZnO catalyst is more favorable than the (0001) facet, as demonstrated by the DFT calculations (Fig. 19g, h) [268]. Experimental results further indicated that ZnO nanoparticles with a high fraction of (10 $\overline{1}$ 0) facets own outstanding performance for catalytic H_2_O_2_ generation: the overpotential of 40 mV at a current density of 0.1 mA cm^−2^ and an FE of 81% during this 2e^−^ WOR process (Fig. 19i). Mn-alloyed TiO_2_ coatings, prepared by atomic layer deposition, also exhibited the high performance of electrocatalytic H_2_O_2_ generation via a 2e^−^ WOR pathway: e.g., an FE of higher than > 90% at the overpotential smaller than 150 mV [269]. It has to highlight here that the principles in designing the electrocatalysts with high performance toward the 2e^−^ WOR is still unclear at this stage. More explorations on reaction mechanism for WOR need be provided in future.

#### Boron-Doped Diamond

As discussed, various noble metal oxides have shown the capability to generate H_2_O_2_ through a 2e^−^ WOR pathway. However, these noble metals are expensive and rare. Up to date, the efficiency of the H_2_O_2_ production via 2e^−^ WOR is low. On the other side, noble metal-free oxides are not adapted to be operated at the high current densities and high potentials that are frequently required for the large-scale applications. Among the materials that can endure the corrosion in such a harsh conditions for water oxidation, some molecular metal porphyrins (e.g., AlTMPyP, SiTPyP, ZnTMPyP, SnTMPyP) [273–276] and carbon fibers [57, 277] are promising and thus have been tried for H_2_O_2_ production via the 2e^−^ WOR pathway, but their performance was still poor.

Boron-doped diamond (BDD), as a unique kind of carbon material with excellent physical–chemical stability [278], has been employed for the H_2_O_2_ generation via the 2e^−^ WOR pathway. Since the H_2_O_2_ production through 2e^−^ WOR on the BDD electrode was reported for the first time in 2003 [279, 280], the catalytic performance of electrochemical H_2_O_2_ formation using synthetic BDD films that were coated on a titanium substrate has stimulated extensive attention [281]. The BDD exhibited a H_2_O_2_ yield of as high as 29.0 mmol dm^−3^ at 3.47 V_RHE_. The H_2_O_2_ production rate was 19.7 μmol min^−1^ cm^−2^. However, the FE was only 28% at 3.17 V_RHE_ and total current densities surpassed 120 mA cm^−2^, suggesting a high energy consumption process. To promote the electrocatalytic H_2_O_2_ generation on BDD via the 2e^−^ WOR pathway as well as to decrease the overpotential at a high applied current density, BBD films were optimized by tailoring their boron content, thickness and crystal size [282]. On the BDD-4 (with the B doping level of 12,600 ppm, film thickness of 2 μm), the biggest FE of 87% was reached and the H_2_O_2_ production rate was 76.4 μmol cm^−2^ min^−1^. This BDD-4 was also superb durable (e.g., electrocatalytic H_2_O_2_ production for 10 h at a constant current density of 200 mA cm^−2^). An electrogenerated chemiluminescence (ECL) system was developed via* in-situ* coreactant production at a BDD electrode [283], where peroxydicarbonate (C_2_O_6_^2−^) was produced by the oxidation of carbonate (CO_3_^2−^), subsequently reacted with water in basic media to form H_2_O_2_. Based on experimental and no-economic examination, the efficiency of anodic H_2_O_2_ production on a BDD electrode was explored in an electrochemical H_2_/H_2_O_2_ process using sodium carbonate as an electrolyte [284]. The calculated FE of producing H_2_O_2_ was to up to 31.7% at 2.90 V (*vs*. SHE) and the corresponding production rate reached 3.93 μmol min^−1^ cm^−2^. This study provided guidance to further implement selective oxidation of water to H_2_O_2_ on a BDD electrode.

Note that great progress has been made in the H_2_O_2_ preparation using photo- and photoelectro-catalytic approaches for wastewater treatment at a pilot scale, even an industrial scale. This is because the efficient H_2_O_2_ production is necessary to guarantee Fenton’s reaction [285, 286]. For example, a carbon-PTFE electro-oxidative system promoted the oxidative power of Fenton and Fenton-like processes based on the enhanced H_2_O_2_ generation with a higher current efficiency [287]. Solar photoelectro-fenton (SPEF) with a BDD anode and a cathode of the (Co, S, P)-decorated multiwalled carbon nanotubes showed the excellent performance for bronopol removal due to the high efficiency of H_2_O_2_ production via the 2e^-^ WOR on a BDD [288].

## Conclusions and Perspectives

This article provides an overview of current advances on photo/electro-catalytic H_2_O_2_ production using different pathways. Different photo/electro-catalysts have been detailed. Compared with traditional preparation methods, such as AQ oxidation, based on fossil resources, the direct preparation of H_2_O_2_ through photo/electro-catalytic processes appears to be more promising in that these processes are simple to be operated, green and safe, sustainable and clean, especially when solar or wind energy is integrated. The used catalyst is the prerequisite and technical core to determine the efficiency of such catalytic H_2_O_2_ generation among these technologies, especially in the application beyond the laboratory. In more detail, many advanced photo/electro-catalysts must be constructed under theoretic guidance, including the strategies of their synthesis, design of their components/structures. Once their photo/electro-chemical properties are explored and discussed, their catalytic H_2_O_2_ production is possible to be boosted. However, the mechanism comprehension of various catalysts for their photo/electro-chemical H_2_O_2_ production is still in the preliminary stage of research, and there is still much room for further improvement.

In the field of the photocatalytic synthesis of H_2_O_2_ by means of either the 2e^−^ ORR pathway or the 2e^−^ WOR process, it is concluded that an ideal catalyst needs simultaneously satisfy good photoresponse (e.g., an appropriate band gap), superior separation capacity of photogenerated carriers (namely electrons and holes), as well as high catalytic ability for the H_2_O_2_ yield. However, the reported photocatalysts up to date still suffer from relatively low activity or selectivity of H_2_O_2_, which remains a great challenge for practical applications. Future studies should propose the innovative ideas for applying cooperative modulation of light adsorption, photogenerated charge separation and catalytic reaction actives in photocatalysts to enhance catalytic H_2_O_2_ production. In these regards, supramolecular semiconductors, including MOFs, covalent organic frameworks (COFs) and conjugated polymers can be added as the co-catalysts to improve the H_2_O_2_ yield. This is because the energy band structures of these supramolecular semiconductors can be optimized. In other words, their light absorption, charge separation and redox capability can be tuned during the photocatalytic H_2_O_2_ production. Another promising strategy is to combine inorganic semiconductors with biomolecules to strengthen the response to the solar light and the selectivity of electrocatalytic H_2_O_2_ production. It must be pointed out that although standard photocatalysts (e.g., g-C_3_N_4_ and TiO_2_ materials) have achieved selective H_2_O_2_ synthesis under visible light irradiation, organic sacrificial agents are usually used to improve the separation of photogenerated electrons and holes in these cases. Such an approach inevitably leads to the contamination of H_2_O_2_. Additional cost is then required to separate and obtain pure H_2_O_2_. Consequently, the strategies based on electrochemical assistance, oil–water phase solution and all-solid-state Z-scheme photocatalytic systems appear to effectively facilitate the photogenerated charge separation. Note that the output of H_2_O_2_ produced by the currently reported catalysts is only at the mM or μM level, too far for large-scale and industrial applications. Porous photocatalysts and/or new photocatalytic reaction systems with enhanced mass transfer rates are needed to boost the formed H_2_O_2_ removal from the active sites and to avoid the H_2_O_2_ decomposition. A deep understanding of the photocatalytic mechanism will open up new opportunities for the design of high efficiency photocatalysts. Various high-quality and *in-situ* characterization technologies (e.g., *in-situ* HRTEM, IR, XANES) are helpful to disclose the distinct dynamic behavior and reaction mechanism of different photocatalytic H_2_O_2_ formation systems. In short, exploring effective strategies to promote the ability to harvest wide solar spectrum, improving the lifetime of photogenerated electrons and holes and propelling the 2e^−^ ORR or water oxidation via the design of advanced photocatalysts have been becoming a hot issue for photocatalytic H_2_O_2_ production.

Like photocatalytic H_2_O_2_ synthesis, electrocatalytic H_2_O_2_ synthesis includes two pathways: 2e^−^ ORR and 2e^−^ WOR processes, accompanying with significant achievements acquired in the past few years. Various electrocatalysts with high selectivity (or Faraday efficiencies) and stability have been developed. However, more efforts are still needed to explore and disclose the mechanisms behind. Take the 2e^−^ ORR process as an example, previous reports have revealed that noble metal-based electrocatalysts (e.g., Pt- and Pd-based electrocatalysts) exhibited decent performance for electrocatalytic H_2_O_2_ production. Unfortunately, owing to their high costs and scarce reserves, it is unrealistic to use them for industrial H_2_O_2_ production. To address this issue, noble-based alloys, noble catalysts with core–shell structures, and porous or single atomic noble metal catalysts are highly desirable for high-performance H_2_O_2_ production. Alternatively, carbon-based materials are recognized as potential ones to noble catalysts for the large-scale H_2_O_2_ generation. Note that pristine carbon is generally inert for H_2_O_2_ generation due to its lack of active sites. Its catalytic performance can be integrated and improved by the modification of surface carbon atoms such as the manufacture of porous structures and defects, the introduction of heteroatom doping and oxygen functionalization. Nevertheless, most carbon electrocatalysts are only applicable for the H_2_O_2_ production under alkaline conditions. Their further research is thus needed to improve catalytic performance in acidic media. Moreover, the hurdle of their instability that is caused by the H_2_O_2_ decomposition as well as their low selectivity over a wide potential range inhibits the practical applications of carbon electrocatalysts. Some new electrochemical and photochemical electrode systems need to be designed and developed. For example, BDD, a robust *sp*^3^ hybridized carbon material, has exhibited extremely high durability for the H_2_O_2_ production in harsh conditions. It is thus highly promising for industrial H_2_O_2_ production once the manufacturing cost of BDD films is dramatically reduced. Moreover, the structure–activity relationship of these electrode systems, especially when they are at nanoscales, is critical for the H_2_O_2_ production. Only on their optimized interfaces, the highly efficient H_2_O_2_ synthesis can be realized. To reduce the H_2_O_2_ decomposition, it is better to construct porous structures or multi-site active centers inside carbon electrocatalysts, which can prohibit the formed H_2_O_2_ from further reaction. One more promising strategy is to couple external means (e.g., ultrasound and microwave) with electrochemical approaches to firm up the H_2_O_2_ desorption from the electrocatalysts. To increase the selectivity of the H_2_O_2_ production, the sizes and exposed planes of the used electrocatalysts should be further optimized. The employment of the 2e^−^ WOR process should be paid more attention as a more effective pathway for the H_2_O_2_ production where no gas phase is involved. As confirmed from theoretical calculations and experimental operations, metal oxides (e.g., BiVO_4_, SnO_2_ and ZnO) tend to generate H_2_O_2_ via the 2e^−^ WOR approach, while others (e.g., IrO_2_, PtO_2_ and RhO_2_) are more favorable for the 4e^−^ WOR approach to produce O_2_. In this context, it is highly necessary to further study the mechanisms of the H_2_O_2_ formation via the 2e^−^ WOR approach. Once advanced metal oxide catalysts are found, electrocatalytic H_2_O_2_ production on these metal oxide electrocatalysts can be further enhanced. It is worth mentioning that BDD presented good ability to produce H_2_O_2_ at high potentials. However, it only exhibited low Faraday efficiency. To utilize diamond for industrial H_2_O_2_ production at high current densities, it is urgently needed to improve its catalytic ability by the formation of its nanostructures or the introduction other catalytic centers (e.g., defects, dopants, other catalysts).

To sum up, recent advances in the design and synthesis of photo/electro-catalysts have improved their activity, selectivity and stability for photo/electro-catalytic H_2_O_2_ production in past years. To further improve performance of photo/electro-catalytic H_2_O_2_ production, the design of advanced catalysts with various functions is still highly necessary. For photocatalysts, two-channel reaction pathways for generating H_2_O_2_ with high efficiency are highly pursued. As for electrocatalysts, their high activity with low cost and strong durability is the key factor moving forward. From a practical application perspective, further understanding the reaction kinetics using theoretical and experimental (especially *in situ* and operando techniques) as well as the design of new reactors for the H_2_O_2_ production at an industrial scale must be conducted in future.
